# Tumor Exosomal HIF2A Induce Peritumoral M2 Macrophages Accumulation to Facilitate Intestinal Invasion in Colorectal Cancer

**DOI:** 10.7150/thno.113190

**Published:** 2025-07-02

**Authors:** Dan Wang, Qingyang Lei, Li Yang, Yachang Huo, Weina Yu, Shasha Liu, Yangfei Duan, Shumin Feng, Zhen Li, Jinbo Liu, Zhenqiang Sun, Weitang Yuan, Lihua Liu, Bin Zhang, Yi Zhang

**Affiliations:** 1 Biotherapy Center and Cancer Center, the First Affiliated Hospital of Zhengzhou University, Zhengzhou, Henan 450052, China.; 2 Department of Anorectal Surgery, the First Affiliated Hospital of Zhengzhou University, Zhengzhou, Henan 450052, China.; 3 Department of Tumor Immunotherapy, Fourth Hospital of Hebei Medical University, Shijiazhuang, China.; 4 Department of Medicine-Division of Hematology/Oncology, Feinberg School of Medicine Northwestern University, Chicago, Illinois 60611, USA.; 5 State Key Laboratory of Esophageal Cancer Prevention & Treatment, Zhengzhou University, Zhengzhou, Henan 450052, China.; 6 School of Life Sciences, Zhengzhou University, Zhengzhou, Henan 450052, China.; 7 Tianjian Laboratory of Advanced Biomedical Sciences, Academy of Medical Sciences, Zhengzhou University, Zhengzhou, Henan 450052, China.; 8 School of Public Health, Zhengzhou University, Zhengzhou, Henan 450052, China.; 9 Zhongyuan Cell Therapy and Immunotherapy Laboratory, Henan Academy of Innovations in Medical Science, Zhengzhou, Henan 450052, China.

**Keywords:** CRC, intestinal invasion, macrophages, exosome, CXCL12/CXCR4

## Abstract

**Background:** Local intestinal invasion of tumor cells often leads to recurrent and refractory colorectal cancer (CRC). However, the driven mechanism is not fully understood.

**Methods:** A total of 145 patients with CRC and 10 patients with intestinal perforations or benign lesions were randomly enrolled. The distribution and clinical relevance of macrophages in different tissues were determined by flow cytometry and immunohistochemistry. PCR screening and RNA-sequencing analysis were used to explore the regulatory mechanisms. The functions of macrophages were further verified using an orthotopic mouse model of *Csf1r^cre^Cxcr4^fl/fl^* mice.

**Results:** Here, we unveil the role of M2 macrophages in local intestinal invasion. M2 macrophages infiltrated more in peritumor tissues than in tumor and normal tissues of CRC patients, which were significantly associated with the tumor local intestinal invasion and recurrence. Macrophage elimination attenuated local intestinal invasion. Mechanistically, CXCL12 is highly expressed in peritumor tissues and recruits monocytes and polarizes them into M2 macrophages by binding to CXCR4. Furthermore, *HIF-2α*-containing exosomes from primary colorectal tumor cells promoted CXCL12 secretion by peritumoral fibroblasts through HIF-2α binding to the CXCL12 promoter, which in turn induced M2 macrophage accumulation and tumor cell invasion. Macrophage-specific deletion of CXCR4 or knockdown of *HIF2A* in colorectal tumor cells reduced M2 accumulation in peritumor tissue and subsequent local invasion of tumor cells.

**Conclusion:** These data reveal a fine-tuned collaborative action between primary cancer cells and peritumoral stromal cells in distinct tumor areas, which reshape the immunosuppressive microenvironment and inducing local intestinal invasion via the HIF2A/CXCL12/CXCR4 axis.

## Introduction

Colorectal cancer (CRC) is a highly fatal gastrointestinal tumor characterized by robust inflammation and tumor cell metastasis. The dissemination of colorectal tumor cells leads high mortality. Studies demonstrated that CRC disseminate in four main ways: local intestinal invasion, lymphatic spread, hematogenic spread, and nerve spread. Among these, local intestinal invasion occurs more frequently and is more lethal in patients with CRC 1, 2. Hence, understanding the mechanisms of local intestinal invasion of CRC may shed light on novel treatment strategies.

Recent evidence suggests that pre-metastatic niches develop prior to primary tumor cell dissemination and support colonization, survival, and growth of tumor cells, thus facilitating distant tumor spread 3. During this process, various types of immune cells contribute to the formation of the stromal environment in the premetastatic niche 4. Macrophages were reported to participate in the initiation of the premetastatic niche in the liver 5. Our previous study revealed that M2 macrophages, an important subset of tumor-associated macrophages (TAMs), promote cancer stemness. High expression of their biomarkers CD163 and CD206 in tumor tissues predicted poor prognosis in glioma and non-small cell lung cancer 6, 7. However, whether the mechanisms in the formation of pre-invasive niches are similar to premetastatic niches are not well understood.

Exosomes are membranous extracellular vesicles (30-150 nm in diameter) that participate in intercellular communication by transporting biochemicals such as cytokines, mRNAs, miRNAs, and proteins. These characteristics can promote the progression of many solid tumors 8, 9. Our recent study showed that exosomal miRNA-21 promotes the progression of esophageal squamous cell carcinoma 10. Recent studies have proposed that exosomes released from primary tumors create a suitable microenvironment for disseminated malignant cells in distant organs 11. However, the mechanisms underlying the promotive effects of exosomes on pre-invasive niches are not yet fully understood.

Hypoxia inducible factor-2α (HIF-2α, corresponding gene name *HIF2A*) plays a pro-tumor role in gastrointestinal tumors and is linked to a worse prognosis 12, but whether HIF-2α is a key pro-invasive factor and can be transferred by exosomes has not been investigated.

In this study, we verified that M2 macrophages gradually increased in the peritumoral intestinal tissues of patients with CRC progression and were associated with intestinal invasion of the primary tumor. We investigated the exosome-mediated molecular interactions between primary tumor cells and stromal cells in peritumor tissues, which promoted the accumulation of M2 macrophages. CXCL12 is one of the mediators of the interaction between primary tumor cells and the stroma, and has been reported to promote tumor progression 13, as well as recruited macrophages by combining with CXCR4 14. Our results showed that primary tumor-derived exosomes loaded with *HIF-2A* recruited M2 macrophages by enhancing CXCL12 secretion from peritumoral fibroblasts and promoting tumor invasion. Inhibiting CXCR4 significantly reduced M2 macrophage accumulation and tumor invasion. Therefore, these data suggest a novel strategy for limiting CRC invasiveness, and disease recurrence.

## Results

### The predominant accumulation of M2 macrophages in peritumor tissue correlates with tumor progression and poor survival in CRC patients

**Figure 1:**
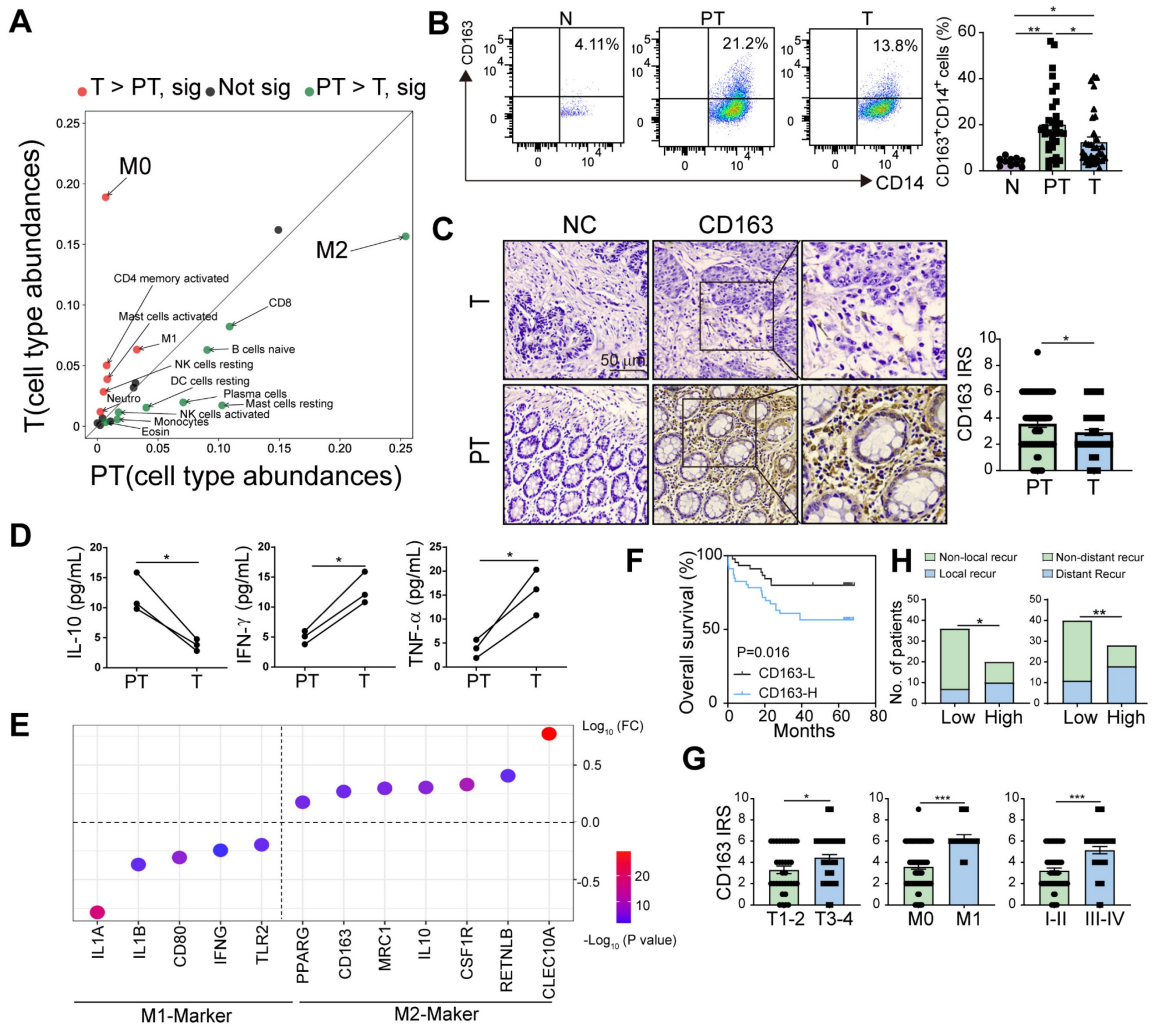
**Higher accumulation of M2 macrophages in peritumor versus tumor tissue correlates with poor survival in CRC patients.​ A.** Relative abundance of 22 types of immune cells in peritumor intestine of patients with CRC analyzed using the CIBERSORT from TCGA database. **B.** The proportions of CD163^+^ M2 macrophages in CD14^+^ cells were detected in normal intestinal tissue (N) from patients with benign lesion or intestinal perforation, tumor tissue (T) and paired peritumor intestinal tissue (PT) from the patients by flow cytometry. **C.** Immunohistochemical profiles of CD163 in PT and T under microscopy (Left). IRS (0-9) = intensity score (0-3) × percentage score (0-3) (Right). **D.** The expression of 13 cytokines in conditioned medium of CD163^+^CD14^+^ M2 macrophages purified from PT and T were analyzed by using multiplex assay. Cytokines with significant differences are listed in the figure. **E.** The expression of M1/M2- related genes in M2 macrophages from PT and T using TCGA datasets. (FC, PT/T). **F.** The relationship between overall survival of the patients and CD163 expression in PT (n = 91). **G.** IRS of CD163 in PT of the patients with different T stages (T1-2, tumor does not reach the subserous membrane; T3-4, tumor reaches or extends beyond the subserous membrane), M stages (M0, non-distant metastasis; M1, distant metastasis) and I-IV stages (I-II, early stage; III-IV, advanced stage). **H.** Histogram showing the numbers of local/distant recurrent and non-local/distant recurrent patients with high and low CD163 levels. The peritumor samples were stratified as 'high' and 'low' according to the median CD163 level (median of CD163 IRS = 4). Recur, recurrent. P values were determined by two-sided Student's t test in panels B, C, D, G or Fisher's exact test in panel H; Data were shown by mean ± s.e.m. * P < 0.05, ** P < 0.01.

To profile the distribution of immune cells in the tumor and peritumor tissues of patients with CRC, we analyzed the relative abundance of 22 immune cell types from The Cancer Genome Atlas (TCGA) using cell-type identification by estimating relative subsets of known RNA transcripts (CIBERSORT) algorithm 15 and found that the abundance of M2 macrophages in the peritumor tissues was higher than in the tumor tissues, whereas the abundance of M0 macrophages showed the opposite trend (Figure 1A). There was no significant difference in CD68 expression (M0 macrophages) on protein level between tumor and peritumor tissues (Figure S1A).

M2 macrophages are critical participants in tumor aggression. We first analyzed the distribution of M2 macrophages in the normal intestine of patients with benign intestinal disease and in tumor tissues with paired peritumor tissues from patients with CRC. The results showed that the percentage of M2 macrophages (CD163^+^CD14^+^) in the peritumor intestine was higher than in the other two groups (Figure 1B), which was later confirmed by immunohistochemical staining for CD163 in CRC tissues (Figure 1C). The consistent trend was also found in the CD163^+^CD68^+^ M2 macrophages (Figure S1B). Similarly, analysis of data from patients with CRC in TCGA and GEO databases showed that *CD14*,* CD163*, and* CD206* expressions were higher in peritumor intestine than in tumor (Figure S1C-F). Furthermore, we isolated the peritumor- and tumor-infiltrating M2 macrophages, and found that M2 macrophages in peritumor secreted significantly higher of IL-10 and lower levels of IFN-g and TNF-α, compared with that in tumor using Multiplex assay (Figure 1D). This was in line with the bioinformatics analysis of TCGA datasets showing that M2 macrophages in the peritumor intestine possess a greater M2- and lesser M1-like signatures (Figure 1E). These results indicate that peritumor-infiltrating M2 macrophages are more immunosuppressive.

To evaluate the prognostic role of peritumoral M2 macrophages, we divided the patients into two groups according to their immunoreactive scores (IRSs) for CD163 and found that high CD163^+^ cell density in peritumor tissues was associated with poor overall survival (OS) (Figure 1F). Moreover, the expression level of CD163 in peritumor tissues was significantly increased in patients with deep invasion, distant metastasis, and advanced-stage (Figure 1G). High expression of CD163 were closely related to high local and distant recurrence rates (Figure 1H). Therefore, we proposed that peritumoral M2 macrophages are closely associated with disease progression.

### M2 macrophages accumulated in pre-invasive intestine promote local intestinal invasion

**Figure 2:**
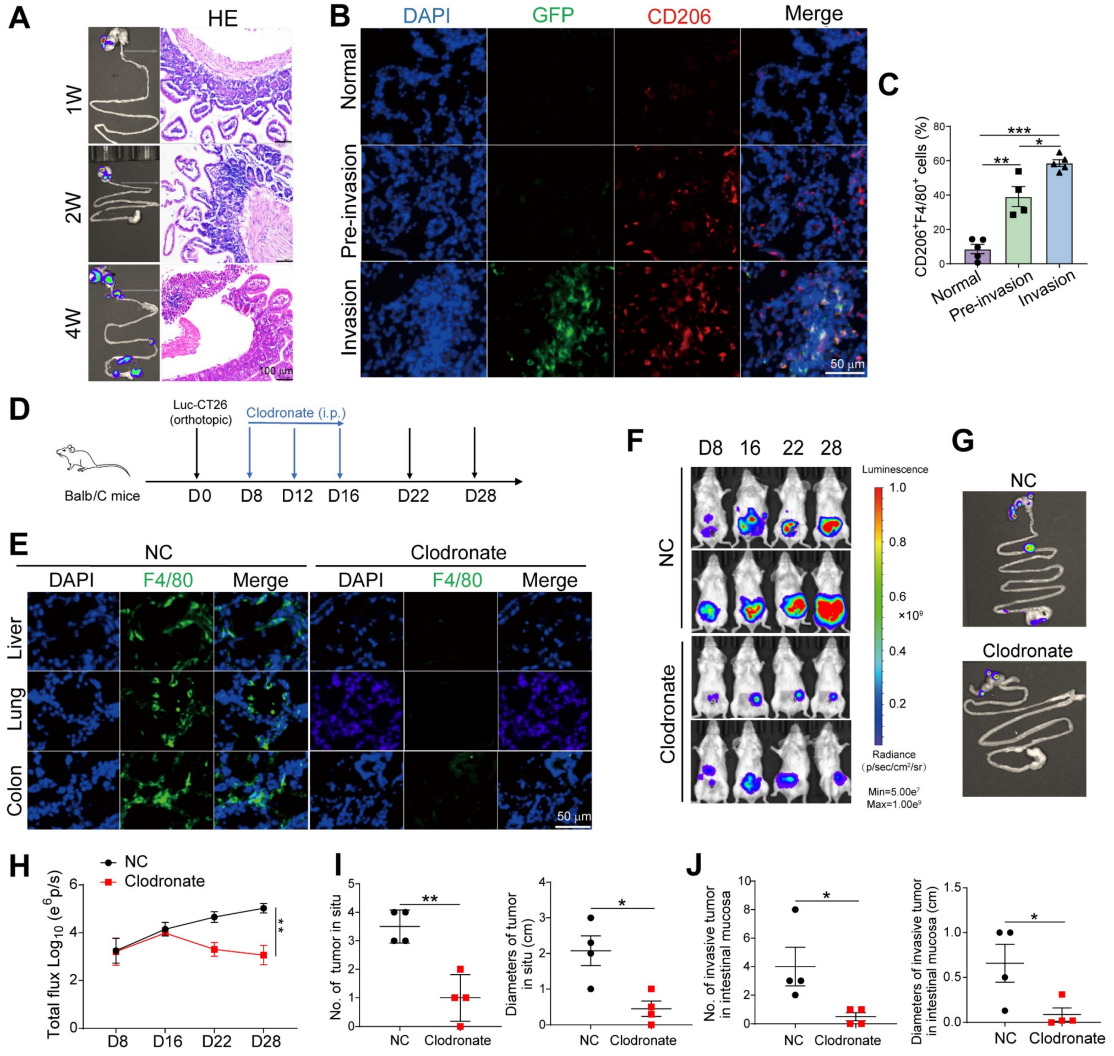
**Macrophage elimination prevents formation of intestinal pre-metastatic niche. A.** Luc-GFP-CT26 cells were injected into the cecal wall of mice at 8 weeks of age. Intestinal invasion in 1-, 2-, and 4-week groups was monitored weekly using *in vivo* bioluminescence imaging (Left). H&E section of peritumor intestine (Right). **B.** Representative images of immunofluorescence (IF) for GFP^+^ tumor cells (green), CD206^+^ M2 macrophages (red) in peritumor intestine of 1-week (normal), 2-week (pre-invasion), and 4-week (invasion) groups. **C.** The proportions of CD206^+^F4/80^+^ M2 macrophages in F4/80^+^ cells were detected by flow cytometry in peritumor intestine.** D.** Schematic illustration for clodronate liposomes administration intraperitoneally at days 8, 12, and 16 after Luc-GFP-CT26 cells were injected orthotopically. Tumor evolution was monitored using *in vivo* bioluminescence imaging. Primary tumor and intestinal invasion were detected at day 28. **E.** The efficiency of macrophage elimination was evaluated by F4/80 staining in the liver, lung, and colon. **F. and G.** Images of primary tumor in the cecum and invasive tumor in the adjacent colon by an* in vivo* imaging system. **H.** Quantitative photon counting analysis of tumor progression by an *in vivo* imaging system. **I and J.** The diameters and numbers of primary and invasive tumors in intestinal tissues were assessed. P values were determined by Two-sided Student's t test in panels C, I, J or two-way ANOVA in panel H; Data were shown by mean ± s.e.m. * P < 0.05, ** P < 0.01, *** P < 0.001.

To verify the effects of peritumoral M2 macrophages, we constructed an orthotopic CRC mouse model by injecting Luc-GFP-CT-26 cells into the cecal walls of BALB/c mice. Primary tumor growth and adjacent intestinal invasion were monitored by bioluminescence and HE staining at weeks 1, 2, and 4. Two weeks after inoculation, we found inflammatory cells in adjacent intestine with no luciferase^+^ tumor cells, which was defined as "pre-invasive" phase (Figure 2A). Luciferase^+^ tumor and inflammatory cells were detected in the adjacent intestine at week 4 of the invasive phase (Figure 2A). Immunofluorescence detection of CD206^+^ macrophages (Figure S1G and 2B) and GFP^+^ tumor cells in the adjacent intestine revealed significantly increased CD206^+^ macrophage infiltration in the tumor-invasive areas compared to both normal tissues and pre-invasive tissues, with the highest levels observed in the invasive regions (Figure 2B). This finding was further confirmed by flow cytometric analysis of CD206^+^ macrophages (Figure 2C). Together, these data suggest that M2 macrophages accumulate in the pre-invasive intestine and are closely associated with local intestinal invasion.

To further evaluate the role of macrophages in intestinal invasion, we depleted macrophages *in vivo* using clodronate liposomes (Figure 2D and E) and found that primary tumor and intestinal invasion were reduced (Figure 2F-H). The number and size of primary and invasive tumors in the peritumor intestine were reduced (Figure 2I and J). Thus, M2 macrophages are critical for promoting preinvasive niche formation.

### Peritumoral fibroblast-derived CXCL12 mediate the accumulation of M2 macrophages in pre-invasive intestinal tissues through binding to CXCR4

**Figure 3:**
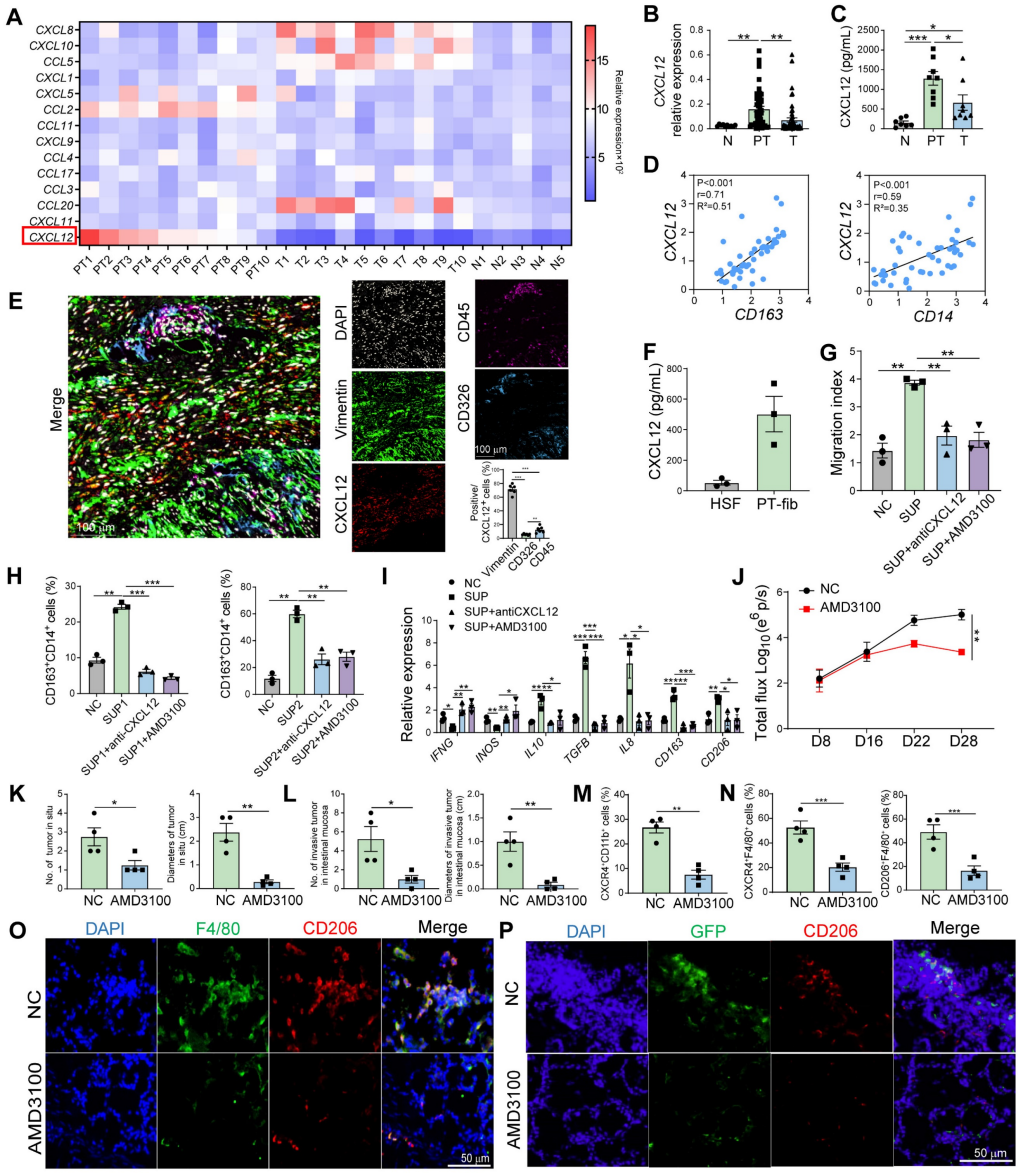
** Peritumoral fibroblast-derived CXCL12 mediate the accumulation of M2 macrophages in pre-invasive intestinal tissues through binding to CXCR4. A.** qPCR screening of immunosuppressive cell-related chemokines expression in N (n = 5), T (n = 10) and PT (n = 10) tissues from patients. **B.** qPCR analysis of *CXCL12* expression in N (n = 10), T (n = 52), and PT (n = 52) tissues from patients. **C.** Quantification of CXCL12 in the supernatants of N, T, and PT tissues from patients by ELISA. **D.** Pearson correlation between *CXCL12* and *CD163/CD14* expression (-Log_10_ of mRNA relative expression) (n = 49). **E.** Representative images of multicolor immunofluorescence identified by co-staining with CXCL12 (red) and CD326^+^ epithelial cells (Cyan) or vimentin^+^ fibroblasts (green) or CD45^+^ immune cells (purple) in the peritumoral areas. The percentages of vimentin^+^ fibroblasts, CD326^+^ epithelial cells, and CD45^+^ immune cells in CXCL12^+^ cells were also evaluated by IF analysis. **F.** Quantification of CXCL12 in the supernatant of human skin fibroblasts (HSFs) and primary fibroblasts derived from PT tissues (n = 3). **G.** Migration of purified CD14^+^ monocytes from healthy donors pretreated with AMD3100 (a CXCR4 inhibitor) and co-cultured with the supernatants (SUP) of primary fibroblasts pretreated with anti-CXCL12 antibody was analyzed using the Transwell assay. Migration index was calculated by dividing the number of cells that migrated to the indicated groups by the number of cells that migrated to the control group. **H and I.** The proportion of CD163^+^ M2 macrophages among CD14^+^ cells and the expression of M1- and M2-related genes were detected using flow cytometry and qPCR. *P < 0.05, **P < 0.01, ***P < 0.001.** J.** AMD3100 was administrated intraperitoneally at 8 days after Luc-GFP-CT26 cells were orthotopically injected. Tumor evolution was monitored using *in vivo* bioluminescence imaging. Primary tumor and intestinal infiltration were detected at day 28. Quantitative photon counting analysis of tumor progression by an *in vivo* imaging system. **K and L.** The diameters and numbers of primary tumor and invasive tumor in intestinal tissues were compared between AMD3100 and PBS treated groups. **M and N.** The proportions of CXCR4^+^CD11b^+^ cells in blood and CXCR4^+^F4/80^+^ cells and CD206^+^F4/80^+^ M2 cells in PT tissues were detected by flow cytometry. **O.** Representative images of immunofluorescence (IF) for F4/80^+^ (green) and CD206^+^ (red) M2 macrophages in peritumor intestine of AMD3100 and PBS treated groups. **P.** The invasion of GFP^+^ tumor cells and infiltration of CD206^+^ M2 macrophages in peritumor tissue were compared between AMD3100 and PBS treated groups. P values were determined by Two-sided Student's t test in panels B, C, F-I, K-N or two-way ANOVA in panel J; Data were shown by mean ± s.e.m. * P < 0.05, ** P < 0.01, *** P < 0.001.

The constant and rapid replenishment of circulating blood monocytes is the primary source of intestinal macrophages 16. Chemokines account for the migration and polarization of macrophages originating from peripheral blood monocytes 17. Therefore, we profiled monocyte-related chemokines from the normal intestine, tumor tissue, and paired peritumor intestines. The results found that *CXCL12* expression in the peritumor intestine was significantly higher than in the normal intestine and tumor tissues (Figure 3A, Figure S2A). We then validated the findings using a larger number of patient samples and found that CXCL12 levels in the peritumor intestine were significantly higher (Figure 3B and C). These results were also confirmed in TCGA dataset, which contains paired and unpaired tumoral and peritumor tissues (Figure S2B and C).

Correlation analysis showed a positive relationship between *CXCL12* expression and *CD14* and *CD163* expression in the peritumor intestine (Figure 3D), indicating that CXCL12 drives the accumulation of M2 macrophages in the pre-invasive niche. Considering that those accumulated macrophages were generated from peripheral blood monocytes, we examined their chemokine receptor expression and found that CXCR4, a receptor for CXCL12, was highly expressed in CD14^+^ monocytes (Figure S2D). Chemotaxis experiments showed that recombinant human CXCL12 recruited CD14^+^ monocytes in a dose-dependent manner, which was inhibited by the CXCR4 inhibitor (AMD3100) (Figure S3A). Furthermore, monocytes cultured in medium containing rhCXCL12 for 7 days exhibited increased size and irregularity, morphological features similar to those of M2 macrophages classically induced by M-CSF (Figure S3B). In addition, CXCL12 stimulation led to an increase in the percentage of CD163^+^ cells (Figure S3C) and in the mRNA levels of M2-related factors (*CD163*, *TGFB*,* IL10*, and *ARG1*), along with a decrease in the mRNA expression of M1-related factors (*IL12A*, *TNFA*, and *IFNG*) (Figure S3D).The role of CXCL12 in polarization was further confirmed by comparing the two M-CSF-cultured groups in the presence or absence of rhCXCL12 (Figure S3E). These data suggest that CXCL12 promoted monocyte migration and polarized monocytes to the M2 phenotype via CXCR4 *in vitro*.

To determine the underlying mechanism, we analyzed several polarization-related pathways and found that the phosphorylation of MAPK, ERK, AKT and P65 increased in a concentration-dependent manner after treatment with rhCXCL12 (Figure S4A). Inhibition of MAPK, ERK, or CXCR4 using SB203580, LY3214996, or AMD3100, respectively, downregulated the percentage of CD163^+^ macrophages and expression of M2-related genes, and rescued the decrease in M1-related gene expression induced by CXCL12 (Figure S4B-D). These results demonstrated that CXCL12/CXCR4/MAPK/ERK signaling is involved in inducing monocyte differentiation into M2 macrophages.

Previous studies have shown that CXCL12 is primarily derived from fibroblasts, epithelial cells and immune cells 18. To determine which type of cells secreted CXCL12 in the peritumor intestine, we detected the expression of CXCL12 in peritumor tissues using multicolor immunofluorescence and found that CXCL12 expression was higher in vimentin^+^ fibroblasts than in CD326^+^ epithelial cells and CD45^+^ immune cells (Figure 3E). CXCL12 expression in the peritumoral intestinal fibroblasts was higher than in the normal fibroblasts (Figure 3F). We found that the supernatants of primary fibroblasts from the peritumor intestines significantly increased the migration of CD14^+^ monocytes, which was attenuated by anti-CXCL12 or AMD3100 treatment (Figure 3G). In addition, peritumoral fibroblast-derived supernatants enhanced the percentage of CD163^+^ macrophages and M2-related factors (*TGFB*, *IL10*, *IL8*, *CD163*, and *CD206*), but decreased the percentage of M1-related factors (*IFNG* and *INOS*) (Figure 3H and I). Consistently, the effect of peritumoral fibroblast-derived supernatants was markedly attenuated by treatment with anti-CXCL12 or AMD3100 (Figure 3H and I). Collectively, these results demonstrate that fibroblast-derived CXCL12 in the peritumor intestine (pre-invasive niche) recruits monocytes from the circulatory system into the peritumor intestine and induces them into the M2 phenotype by binding to CXCR4.

### Inhibition of CXCR4 reduces intestinal invasion

Immunofluorescence analysis revealed higher CXCR4 expression in M2 macrophages within peritumoral tissues compared to tumor tissues (Figure S5A and B). Similarly, we observed higher CXCR4 expression in purified M2 macrophages from peritumor tissues versus tumor tissues (Figure S5C). To further elucidate the role of CXCR4 in macrophage migration and intestinal invasion *in vivo*, we purified CXCR4^+^CD11b^+^ and CXCR4^-^CD11b^+^ cells from mouse bone marrow and transferred them intravenously into an orthotopic CRC mouse model 8 days after Luc-GFP-CT26 injection (Figure S6A). Results showed that CXCR4^+^CD11b^+^ cell transfer promoted primary tumor growth and intestinal invasion (Figure S6B-E), indicating CXCR4^+^ monocytes promoted tumor intestinal invasion.

To evaluate whether CXCR4 blockade rescued M2 macrophage-mediated intestinal invasion, we administered AMD3100 intraperitoneally at 8, 12, and 16 days after the orthotopic injection of Luc-GFP-CT26 cells. The CXCR4 blockade significantly suppressed primary tumor growth (Figure 3J). The number and size of the primary and invasive tumors also decreased (Figure 3K and L). In addition, in the AMD3100 treated group, the proportion of CXCR4^+^CD11b^+^ cells in the peripheral blood and the infiltration of CXCR4^+^F4/80^+^ macrophages in peritumor tissues decreased (Figure 3M and N). The proportion of CD206^+^F4/80^+^ M2 macrophages in the peritumor intestine decreased dramatically when CXCR4 expression was blocked (Figure 3O). Moreover, the invasiveness of GFP^+^ tumor cells in the peritumor intestine was significantly reduced (Figure 3P). These data indicate that CXCR4 blockade disturbs M2 macrophage accumulation in the peritumor intestine, which can inhibit intestinal invasion.

### *HIF2A* in exosomes from primary tumor cells upregulate CXCL12 expression in peritumoral fibroblasts

According to our results, M2 macrophage infiltration was higher in the peritumor intestine than in the normal intestine (Figure 1B), indicating that primary tumors affect macrophage infiltration in the pre-invasive intestine. Consistently, peritumor-derived fibroblasts expressed higher CXCL12 than normal fibroblasts (Figure 3F). However, the mechanism of primary tumor-induced CXCL12 expression in the peritumor intestine remains unknown.

**Figure 4:**
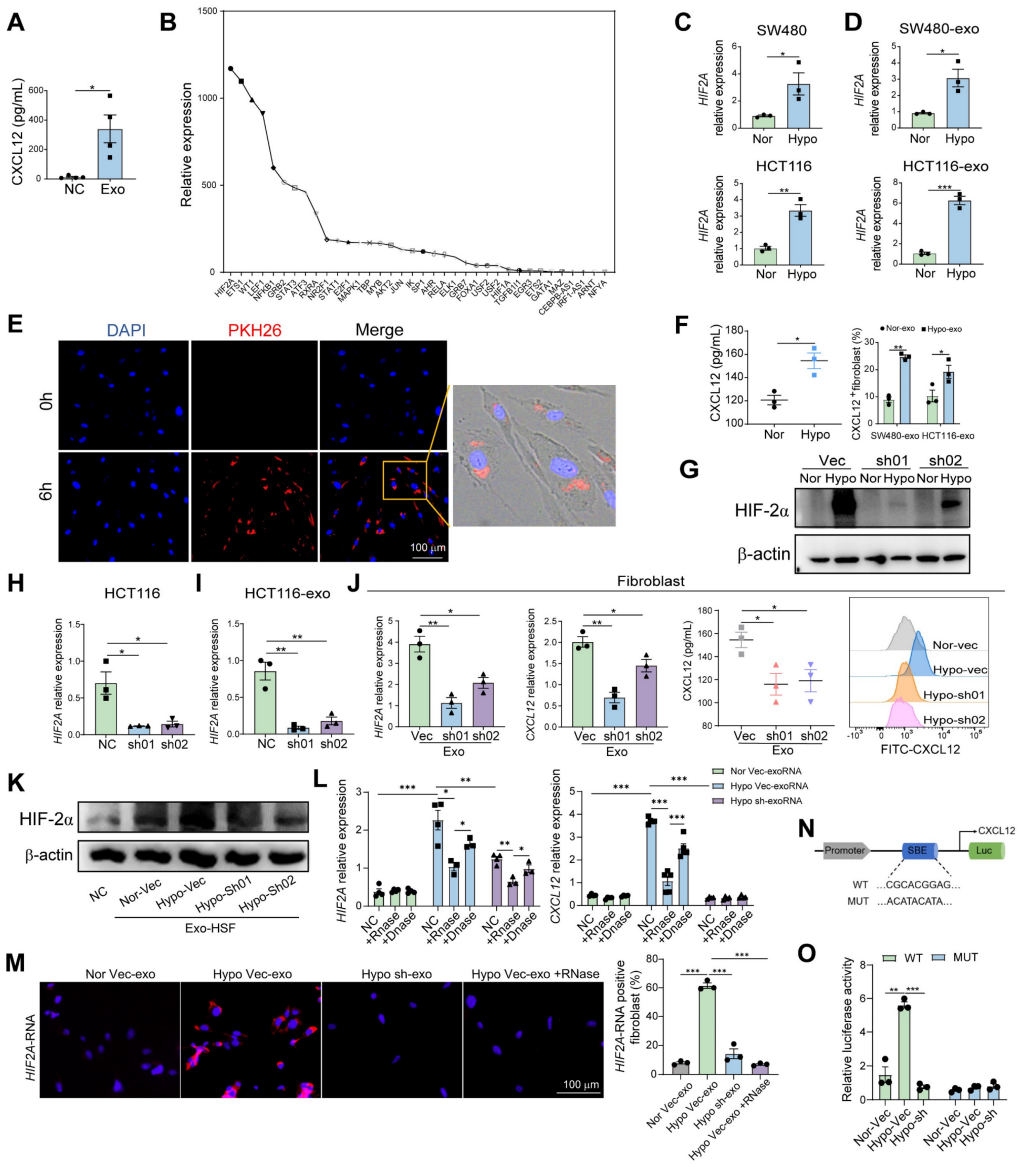
**HIF2A-expressed exosomes derived from primary tumor cells induced CXCL12 secretion by peritumoral fibroblasts. A.** Quantification of CXCL12 in supernatant of peritumor-derived primary fibroblasts treated with or without SW480 cell-derived exosomes. **B.** The upstream transcriptional factor of *CXCL12* was identified via the PROMO website. The relative expressions of the upstream transcriptional factors in exosomes collected from conditioned medium of HCT116 cells using RNA-seq are presented in the graph. **C and D.** qPCR analysis of *HIF2A* expression in SW480 and HCT116 cancer cell lines (C), SW480- and HCT116-derived exosomes (SW480-exo and HCT116-exo) (D) under normoxic (21% O_2_) and hypoxic (1% O_2_) culture. (Nor: normoxia, Hypo: hypoxia) **E.** Microscopy image of the internalization of fluorescently labelled exosomes (PKH26) in fibroblasts. **F.** Quantification of CXCL12 in supernatant of peritumor-derived primary fibroblasts treated with HCT116-derived exosomes under normoxic and hypoxic culture by ELISA experiment (Left). The proportions of CXCL12^+^ cells in fibroblasts treated with or without SW480-exo or HCT116-exo were detected by flow cytometry (Right). **G.** HIF-2α was examined by western blotting in *HIF2A*-silenced HCT116 cells (sh01 and sh02) and control cells (Vec: vector) under normoxic and hypoxic cultures. **H.** *HIF2A* was examined by qPCR in *HIF2A*-silenced HCT116 cells and Vector-HCT116 cells. **I.** *HIF2A* was examined by qPCR in exosomes from *HIF2A*-silenced HCT116 cells and control cells. **J.** *HIF2A* mRNA and CXCL12 expression were evaluated in primary fibroblasts treated with or without Sh*HIF2A*-HCT116- or Vector-HCT116-derived exosomes under hypoxic condition. **K.** HIF-2α expression on protein level in HSF under hypoxia treated with or without exosome derived from sh*HIF2A*-HCT116 and control cells under normoxic and hypoxic cultures. **L.** mRNA expression of *HIF2A* and *CXCL12* in the HSF cells stimulated with tumor cell exosomal RNAs (exoRNA), pretreated with or without RNase or DNase.** M.** *HIF2A* RNA detection in HSF using FISH probes (blue: DAPI nuclear staining; red: *HIF2A* RNA).** N.** The position of the putative HIF-2α binding element (SBE) in the human CXCL12 promoter. Sequences of the wild-type (WT) and mutated (MUT) binding element in the luciferase reporter constructs. **O.** The relative luciferase activity of the WT or Mut luciferase reporters was detected in 293T cell or sh*HIF2A*-293T cell under normal and hypoxic conditions. P values were determined by two-sided Student's t test in panels A, C, D, F, H-J, L, M, O; Data were shown by mean ± s.e.m.* P < 0.05, ** P < 0.01, *** P < 0.001.

Exosomes are small vesicles that transfer diverse proteins, RNAs, and DNAs from primary tumors to pre-metastatic organs 19**.** Therefore, we first purified the tumor cell-derived exosomes and verified them using NTA (NanoSight) and electron microscopy (Figure S7A and B), then examined the exosome-expressed proteins (CD9 and TSG101) using WB (Figure S7C). To determine the relationship between exosomes and CXCL12, we co-cultured peritumor-derived fibroblasts with SW480 cell-derived exosomes and found that the secretion of CXCL12 was significantly increased (Figure 4A). We then used the PROMO website to search for molecules upstream of CXCL12 and identified 36 possible transcription factors that account for CXCL12 expression. RNA-sequencing (RNA-seq) of HCT116 cell-derived demonstrated the highest expression level of *HIF2A* (Figure 4B). Therefore, we postulated that exosomes transfer *HIF2A* to peritumor-derived fibroblasts to induce CXCL12 production. Given that hypoxic environment induces *HIF2A* expression, we cultured SW480 and HCT116 cells under hypoxic conditions and detected an upregulation of *HIF2A* mRNA in their exosomes (Figure 4C and D). We found that PKH26-labeled exosomes from SW480 cells entered the peritumoral fibroblasts (Figure 4E) and enhanced the protein level of CXCL12 (Figure 4F). To definitively confirm that exosomes deliver *HIF2A* mRNA, but not protein, to regulate CXCL12 expression, we generated *HIF2A* knockdown (KD) and overexpression (OE) HCT116 cell lines. First, western blot analysis revealed that HIF-2α protein was detectable in tumor cells cultured under hypoxic conditions. *HIF2A* shRNA effectively downregulated HIF-2α expression (Figure 4G), whereas OE-*HIF2A* cells increased HIF-2α protein levels (Figure S8A). However, the HIF-2α protein content within exosomes remained below the detection limit of western blotting (Figure S9). Therefore, we hypothesized that exosomes primarily transmit *HIF2A* mRNA rather than HIF-2α protein. We then detected *HIF2A* mRNA in tumor cells and their exosomes. HIF2A knockdown in HCT116 cells significantly reduced, while HIF2A overexpression elevated, *HIF2A* mRNA expression in both tumor cells and their exosomes (Figure 4H and I, Figure S8A and B). Meanwhile, the level of *HIF2A* mRNA (Figure 4J) decreased in primary fibroblasts treated with exosomes from sh*HIF2A*-HCT116 under hypoxic conditions. This was followed by a decrease in its protein level (Figure 4K) and a decrease in the downstream CXCL12 expression (Figure 4J). On the contrary, primary fibroblasts or Human Skin Fibroblasts (HSFs) treated with OE-*HIF2A*-HCT116-derived exosomes showed the opposite trend (Figure S8C and D). Furthermore, to clarify that HIF2A communication in exosomes is mainly through the mRNA form, we isolated tumor cell-derived exosomes, pre-treated with RNase or DNase, and added them into the culture system of HSFs in the presence of RNase inhibitor. The expression of *HIF2A* and *CXCL12* mRNA in HSFs was enhanced after hypoxic Vector-HCT116-derived exosomal RNA (exoRNA) stimulation, which could be blocked by RNase treatment but not by DNase treatment (Figure 4L). However, normoxic Vector-HCT116-derived exosomal RNA and hypoxic sh*HIF2A*-HCT116-derived exosomal RNA had no effect on these gene expressions (Figure 4L). Finally, to elucidate the localization and expression of *HIF2A* mRNA in HSF cells, we performed fluorescence in-situ hybridization (FISH). The results showed that *HIF2A* mRNA was mainly located in the cytoplasm of fibroblasts (Figure 4M). The fluorescence of *HIF2A* mRNA in fibroblasts was higher after culture with hypoxic Vector-HCT116-derived exosomes and lower when treated with hypoxic shHIF2A-HCT116-derived exosomes (Figure 4M). Pretreatment of RNase also reduced the *HIF2A* mRNA signal after hypoxic Vector-HCT116-derived exosome incubation (Figure 4M). Inhibition of lysosomal degradation (with chloroquine, CQ) had little effect on *HIF2A* mRNA expression in fibroblasts treated with OE-*HIF2A*-HCT116-derived exosome (Figure S8E). These findings indicate that tumor cell-derived exosomes carried *HIF2A* mRNA, which further induces the increase of *HIF2A* mRNA and protein levels, and the downstream CXCL12 expression in the recipient fibroblasts.

To clarify whether M2 macrophage accumulation mainly depends on exosomes or HIF2A, we treated fibroblasts with HCT116-derived exosomes or sh*HIF2A* HCT116-derived exosomes and then collected the supernatant to incubate with CD14^+^ cells directly or indirectly. The results showed that the supernatant from HCT116-derived exosome-treated fibroblasts significantly recruited CD14^+^ monocytes and induced M2 macrophage polarization. However, sh*HIF2A* HCT116-derived exosome (Figure S10).

Moreover, luciferase reporter assays revealed that HIF-2α binds to the CXCL12 promoter. We identified a conserved putative HIF-2α binding element in the CXCL12 promoter (Figure 4N) and generated a mutant construct by disrupting this site. Hypoxia induced luciferase activity in wild-type transfectants, whereas HIF2A silencing abolished this induction. In contrast, mutant constructs showed no hypoxia-responsive activity (Figure 4O), confirming HIF-2α as a transcriptional regulator of CXCL12. This direct interaction was further validated by ChIP-qPCR (Figure S11).

### *HIF2A* knockdown in tumoral exosomes inhibits intestine pre-invasive intestine initiation

**Figure 5:**
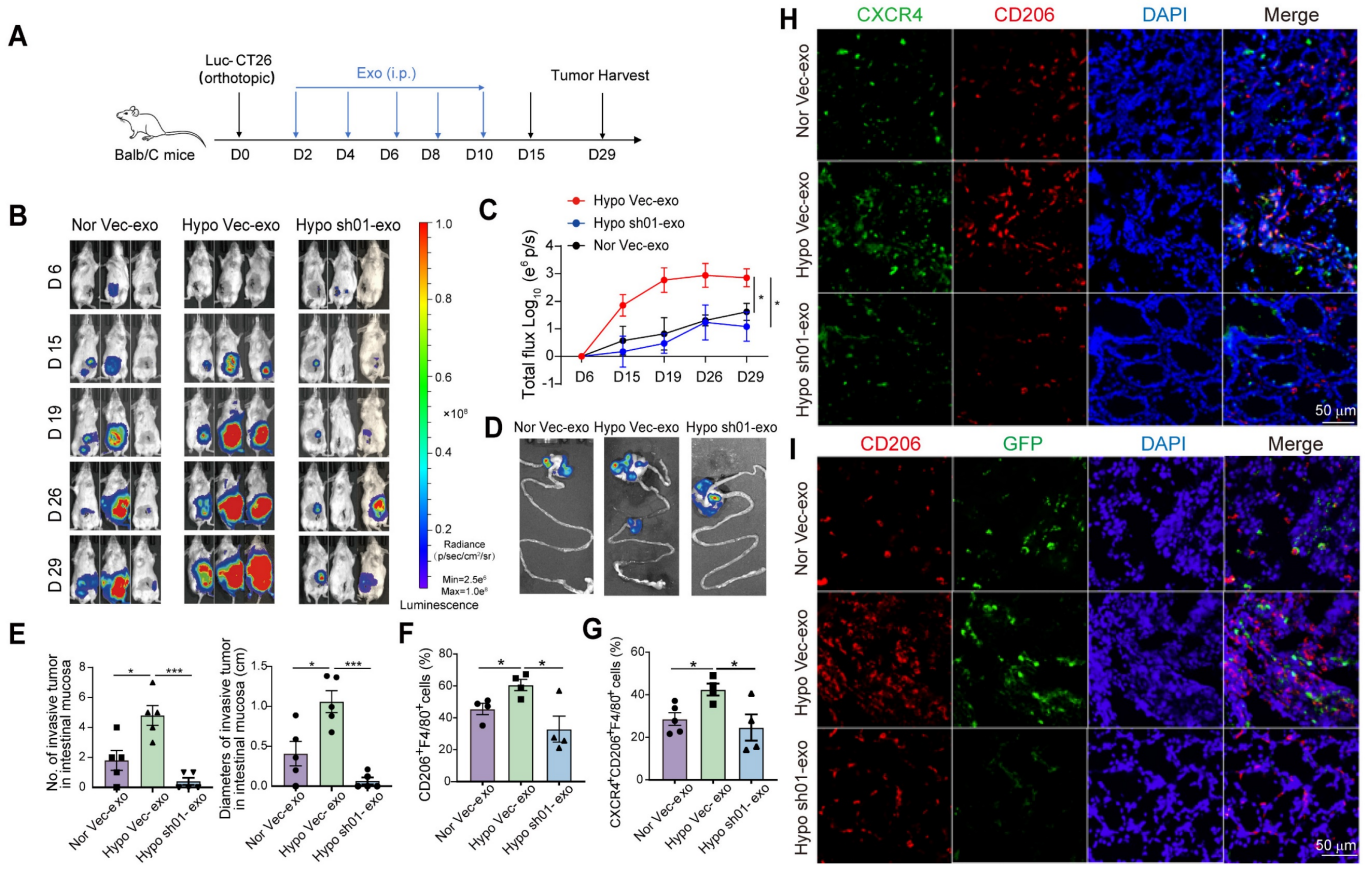
***HIF2A* knockdown in primary tumor exosomes inhibits intestinal pre-invasive niche formation. A.** Schematic illustration for exosome administration intraperitoneally after Luc-GFP-CT26 cells were injected orthotopically. At day 2, 4, 6, 8, and 10, mice were treated with sh*HIF2A*-HCT116-derived exosome under hypoxic culture or Vector-HCT1166-derived exosome under normoxic and hypoxic culture. **B and C.** Quantitative photon counting analysis of tumor progression by an *in vivo* imaging system. Nor vec-exo derived from HCT116 cells that transfected with empty vector and were cultured under normoxia condition; Hypo vec-exo derived from HCT116 cells transfected with empty vector and cultured under hypoxia condition; Hypo sh01-exo derived from *HIF2A*-silenced HCT116 cells under hypoxic condition. **D.** Images of primary tumor in the cecum and invasive tumor in the adjacent colon by an* in vivo* imaging system.** E.** The diameters and numbers of invasive tumor in the intestinal tissues were compared among groups. **F and G.** The infiltration of CD206^+^F4/80^+^ macrophages and CXCR4^+^ CD206^+^F4/80^+^ macrophages in peritumor tissue were evaluated by flow cytometry. **H.** Representative images of IF for CXCR4^+^ (green) and CD206^+^ (red) M2 macrophages in peritumor intestine. **I.** The invasion of GFP^+^ tumor cells and infiltration of CD206^+^ M2 macrophages in peritumor tissue were detected by IF. P values were determined by two-way ANOVA in panel C, or two-sided Student's t test in panel E-G; Data were shown by mean ± s.e.m.* P < 0.05.

To analyze the role of HIF2A in primary CRC cell-derived exosomes on CRC progression, we administered HCT116-derived exosomes to tumor-bearing mice. Following orthotopic implantation of Luc-GFP-CT26 cells, mice received exosome treatments on days 2, 4, 6, 8, and 10 post tumor inoculation (Figure 5A). Hypoxic HCT116-derived exosomes significantly enhanced primary tumor growth, intestinal invasion (Figure 5B-D), and increased the number and size of primary/invasive tumors (Figure 5E). Conversely, *HIF2A*-knockdown exosomes suppressed these effects (Figure 5B-E). Mice treated with hypoxia-derived exosomes showed increased infiltration of CD206⁺ and CXCR4⁺CD206⁺ macrophages, while *HIF2A*-knockdown exosomes attenuated this response (Figure 5F-H). GFP⁺ tumor cell infiltration was similarly modulated (Figure 5I). These findings demonstrate that *HIF2A*-knockdown in CRC-derived exosomes inhibits pre-invasive niche formation.

### Tumoral exosomes promote local intestinal invasion through HIF2A-CXCL12/CXCR4-dependent way

**Figure 6:**
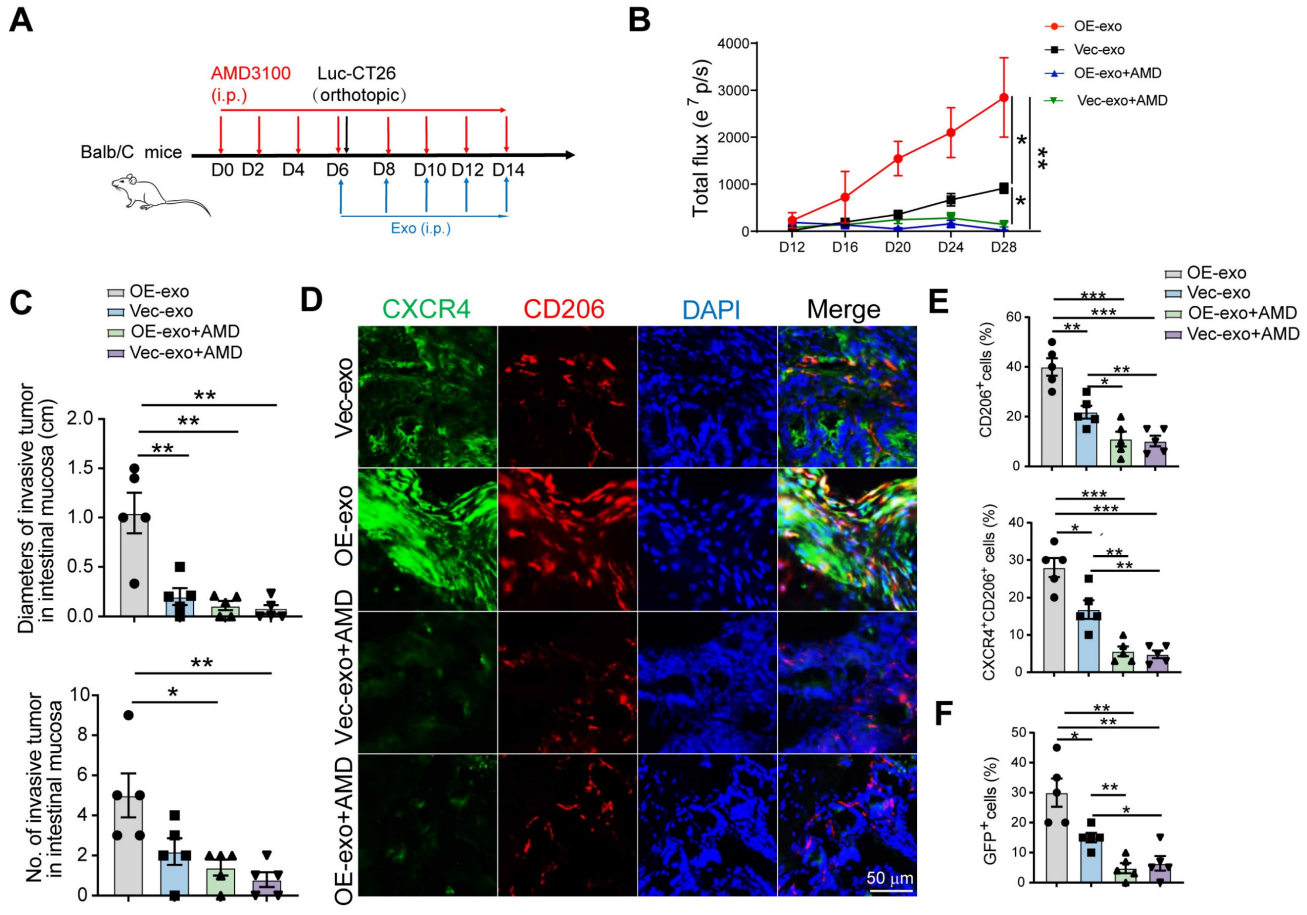
** Tumor exosomes carrying *HIF2A* promote intestinal invasion through CXCL12/CXCR4-mediated M2 macrophage accumulation* in vivo*. A.** Schematic illustration for AMD3100 administration intraperitoneally 6 days before Luc-GFP-CT26 cells were injected orthotopically. At days 8, 10, and 12 after cancer cell implantation, mice were treated with PBS, OE-*HIF2A*-, or Vector-HCT116-derived exosome under normoxic and hypoxic culture intraperitoneally. **B.** Quantitative photon counting analysis of tumor progression by an *in vivo* imaging system. **C.** The diameters and numbers of invasive tumor in intestinal tissues were assessed. **D and E.** Representative images of IF for CXCR4^+^ (green) and CD206^+^ (red) M2 macrophages in peritumor intestine. CD206^+^ and CXCR4^+^ CD206^+^ cell percentage were evaluated by IF analysis. **F.** The invasion of GFP^+^ tumor cells in peritumor tissue were evaluated. P values were determined by two-way ANOVA in panel B, or two-sided Student's t test in panel C, E, F; Data were shown by mean ± s.e.m.* P < 0.05, ** P < 0.01, *** P < 0.001.

Next, we sought to verify whether CRC exosomes promoted pre-invasive niche formation through the HIF2A/CXCL12/CXCR4 axis *in vivo*. Mice were pretreated with AMD3100 for 5 days, followed by the orthotopic injection of Luc-GFP-CT26 cells. AMD3100 and exosomes were intraperitoneally injected every 2 days for an additional week (Figure 6A). *HIF2A*-overexpressing exosomes increased primary tumor growth and invasion, which was inhibited by AMD3100 (Figure 6B-C). Moreover, AMD3100 administration attenuated the proportions of CD206^+^ and CXCR4^+^CD206^+^ macrophages and GFP^+^ tumor cells in the *HIF2A*-overexpressing exosome-treated group (Figure 6D, E and F).

**Figure 7:**
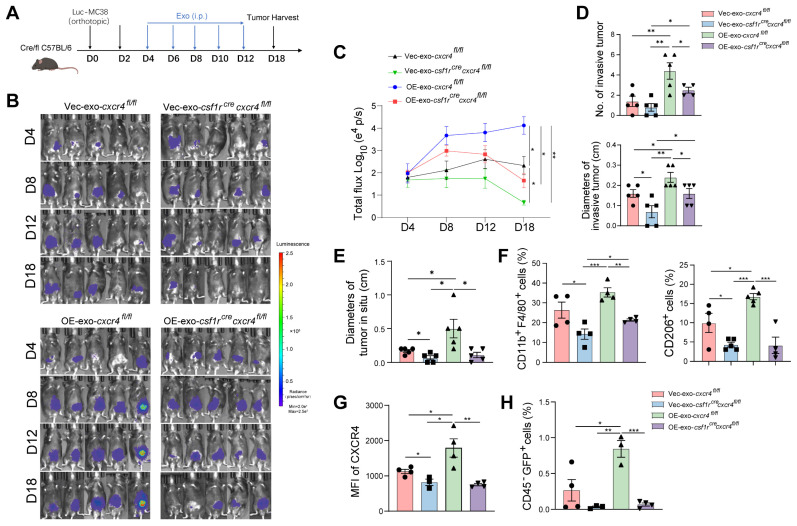
** Macrophage-specific *Cxcr4* knockout in mice inhibits M2 macrophage accumulation and tumor cell invasion stimulated by tumor-derived *HIF2A*-overexpression exosome. A.** Schematic illustration of *Csf1r^Cre^cxcr4^fl/fl^* mice administrated orthotopically with Luc-GFP-MC38 cells. At days 4, 6, 8, 10, and 12 after cancer cell implantation, mice were treated with OE-*HIF2A*- or vector-HCT116-derived exosome. **B and C.** Images and quantitative photon counting analysis of tumor progression by an *in vivo* imaging system. **D and E.** The diameters and numbers of primary tumor and invasive tumor in intestinal tissues were assessed. **F-H.** The invasion of GFP^+^ tumor cells and infiltration of macrophages, M2 macrophages and CXCR4^+^ macrophages in peritumor tissue were evaluated by flow cytometry. P values were determined by two-way ANOVA in panels C, or two-sided Student's t test in panel D-H; Data were shown by mean ± s.e.m.* P < 0.05, ** P < 0.01, *** P < 0.001.

To explore the role of CXCR4 in macrophages during tumor invasion, we used macrophage-specific *Cxcr4* knockout (*Csf1r^Cre^Cxcr4^fl/fl^*) mice to generate an MC38-orthotopic CRC model (Figure 7A). Primary tumor growth and invasion were inhibited in *Csf1r^Cre^Cxcr4^fl/fl^* mice compared to their littermate without *Csf1r*-dependent Cre expression (*Cxcr4^fl/fl^* mice) (Figure 7B-E). Intraperitoneally injected *HIF2A*-overexpressing exosomes promoted primary tumor growth and tumor invasion compared to control exosome injection. *Cxcr4* knockout in macrophages significantly inhibited primary tumor growth and invasion (Figure 7B-E). Furthermore, the infiltration of F4/80^+^ macrophages, M2 and CXCR4^+^ macrophages in peritumor intestine, which was stimulated by *HIF2A*-overexpressed exosome, was also significantly decreased (Figure 7F and G), as was the attenuation of tumor cell infiltration (Figure 7H). Taken together, primary CRC-derived exosomes containing *HIF2A*-stimulated M2 macrophage accumulation through the CXCL12/CXCR4 axis, leading to intestinal invasion.

### HIF2A/CXCL12/CXCR4 expression in peritumor tissue associated with M2 macrophage infiltration and predicted poor prognosis for patients with CRC

**Figure 8:**
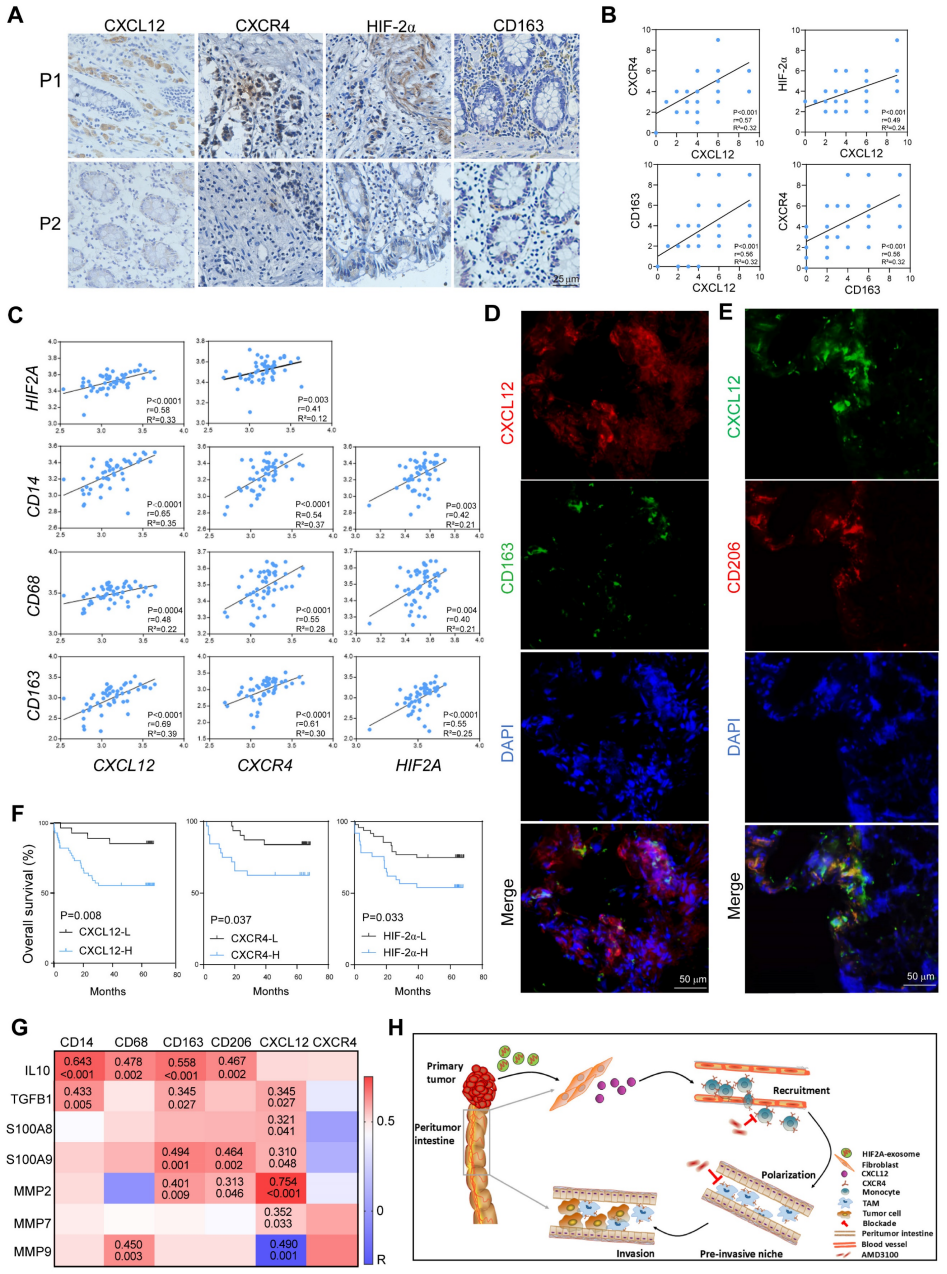
** HIF2A, CXCL12, and CXCR4 expressions in peritumor tissue is associated with M2 macrophage infiltration and predicts poor prognosis in patients with CRC. A.** Expressions of HIF-2α, CXCL12, CXCR4, and CD163 in PT tissues in patients with CRC (P1, a CRC patient with local recurrence; P2, a CRC patient without local recurrence) were immunohistochemically detected. **B.** The Pearson correlation plots were generated by analyzing the IRS of HIF-2α, CXCL12, CXCR4, CD163 (n = 66). **C.** Correlations of gene expressions in peritumor tissue of patients with CRC were analyzed using data from TCGA (n = 50). **D.** Representative images of IF for CD163^+^ (Green) cells and CXCL12^+^ (red) cells in peritumor intestine of patients with CRC. **E.** Representative images of IF for CD206^+^ (Red) cells and CXCL12^+^ (Green) cells in peritumor intestine of the patients. **F.** Kaplan-Meier survival curves for CRC patients (n = 85) with lower and higher IRS of HIF-2α, CXCL12 and CXCR4 expressions (immunohistochemistry analysis). **G.** Correlations between the expressions of CXCL12, CXCR4, M2-related phenotypic markers and the expressions of M2-related functional molecules associated with tumor invasion and metastasis in peritumor tissue from TCGA dataset (n = 50).** H.** Schematic of tumor-derived *HIF2A* carrying exosomes-mediated intestinal fibroblast CXCL12 activation and M2 macrophage accumulation in promoting pre-invasive niche formation.

We utilized the peritumor tissues of patients with CRC to evaluate the effect of HIF-2α/CXCL12/CXCR4/CD163 in the clinic and found that these molecules were expressed higher in the peritumor tissues of CRC patients with local recurrence (P1) than in those without local recurrence (P2) (Figure 8A), indicating their roles in promoting invasion. Consistently, the expression levels of CXCL12 and CXCR4 in the peritumor tissue were significantly increased in patients with advanced-stage disease, distant metastasis, and deep invasion (Figure S12A and B). The HIF-2α expression in the peritumor tissues was lower than in the paired tumor tissues but higher than in the normal tissues (Figure S13). Moreover, our findings indicated that both CXCL12 and CD163 exhibit positive correlations with CXCR4/HIF-2α and CXCL12/CXCR4, respectively, within the peritumor tissue samples (Figure 8B). This was in line with the bioinformatic analysis using TCGA datasets, which showed that the expression of the signaling molecules *CXCL12*, *CXCR4*, and *HIF2A* was positively related to *CD14*, *CD68*, and *CD163*, respectively (Figure 8C). Significantly, we observed that M2 macrophages, characterized by CD163 or CD206 positivity, distributed close to CXCL12^+^ stromal cells. This spatial relationship suggests that these cells play a role in the formation of a pre-invasive intestine (Figure 8D and E). Survival analysis revealed reduced overall survival in patients with CRC with higher HIF-2α, CXCL12, or CXCR4 expressions in peritumor tissues (Figure 8F). Furthermore, using TCGA datasets, we performed correlation analysis between the expression levels of CXCL12, CXCR4, M2 macrophage-associated molecules (*IL10*,* TGFB1*), and tumor invasion-associated molecules (*S100A8*,* S100A9*,* MMP2*,* MMP7*,* MMP9*) in peritumor tissues. The results found that they were positively related (Figure 8G). These data suggest that increased M2 macrophage infiltration and high expression of the signaling molecules CXCL12/CXCR4/HIF2A, predicted intestinal invasion and poor prognosis in patients with CRC.

In summary, primary CRC-derived exosomes, carrying *HIF2A-*mRNA, stimulated peritumoral fibroblasts to secrete CXCL12, which promoted M2 macrophage accumulation and CRC invasion. Therefore, targeting the HIF2A/CXCL12/CXCR4 signaling axis could be an effective therapeutic strategy for CRC (Figure 8H).

## Discussion

In this study, we proposed an innovative mechanism for the local invasion of CRC. We found that exosomes containing *HIF2A-*mRNA released from primary tumor cells stimulate CXCL12 secretion in peritumor fibroblasts, which recruits M2 macrophages to peritumor tissues and promotes tumor invasion. These findings reveal a fine-tuned collaborative action between primary cancer cells and peritumoral stromal cells in tumor microenvironments that are closely associated with CRC progression.

In contrast to primary tumor tissues, peritumor tissues possess abundant immune cell infiltration and complex properties 20, 21. Recent studies have shown that IL-17-producing cells and heterogeneous T cell subpopulations are enriched in the peritumoral stroma of diverse tumor types, which is associated with a poor prognosis 20, 22. Reportedly, M2 macrophages accumulate in the tumor tissues of patients with lung, pancreatic, and gastric cancers and predict poor prognosis 23-25. However, the distribution of M2 macrophages in tumors and peritumor tissues remains controversial. Zheng et al. reported that in patients with hepatic carcinoma, M2 macrophages are highly expressed in peritumor tissues and promote tumor progression 26, which is consistent with our results. In our study, we observed that M2 macrophages primarily infiltrated the peritumoral stroma and were associated with a poor prognosis and disease progression. In Figure 1A, M0 macrophage expression was higher in tumor tissues than in peritumor tissues, as determined by the CIBERSORT algorithm using TCGA data. However, when we confirmed the protein levels of M0 macrophages (identified by CD68 positivity 27, 28) using immunohistochemistry, the results showed no significant difference. It should be noted that the immune cell subtypes were identified based on the gene expression signature specific to each subtype in Figure 1A, which may cause inconsistencies with protein levels.

M2 macrophages mainly accumulate in the peritumoral stroma of patients with CRC, but their nature and underlying mechanisms remain largely unknown. Emerging evidence has revealed that chemokines participate in the accumulation and polarization of M2 macrophages in bone marrow. Studies have been shown that the CCL2/CCR2 axis play an important role in macrophage recruitment 29, 30. Whereas, in our study, we compared the fold changes of the chemokines' mRNA relative expression in peritumor tissues vs tumor tissues and the CXCL12 was most highly expressed in peritumor tissues. The fold change of *CXCL12* was more than 3 times that of *CCL2*. Therefore, we focused on the CXCL12. CXCL12, derived from mesenchymal stromal cells, fibroblasts, and epithelial cells, is associated with poor prognosis in CRC patients 13. CXCL12 promotes the CXCR4-mediated recruitment and chemotaxis of macrophage 14. However, whether M2 macrophages and the CXCL12/CXCR4 pathway play dominant roles in inducing local invasion in CRC remains unexplored. Our study showed that CXCL12 was mainly co-localized with peritumoral fibroblasts and that CXCL12 could recruit monocytes and polarize monocytes into M2 macrophages through CXCL12/CXCR4/MAPK/ERK signaling. Moreover, using an adoptive cell transfer model and CXCR4 blockade, *in vivo* experiments confirmed that the accumulation of peritumoral M2 macrophages mainly depends on the CXCL12/CXCR4 axis. This result is supported by a recent report that CXCL12 promotes M2 macrophage accumulation in lung cancer 31.

M2 macrophage enrichment has been observed in the perivascular niche of tumor tissues and has been shown to promote angiogenesis, inhibit antitumor immunity, and induce cancer stemness 32. However, little is known about the role of M2 macrophages in peritumoral regions. Using bioinformatics analysis and clinical specimens, we found that peritumoral macrophages expressed higher anti-inflammatory factor IL-10 and lower pro-inflammatory factor IFN-g/TNFa than macrophages in tumor tissues, and were associated with local invasion, tumor recurrence, and metastasis. Furthermore, our *in vivo* findings indicated that a large number of M2 macrophages were enriched in the pre-invasive niche and that the primary tumor and local invasive lesions were blocked after macrophage clearance. Preclinical evidence indicates that macrophages interact with tumor cells via inflammatory factors and metalloproteinase (MMP) 33. Our research also showed that peritumoral macrophages were positively related to *IL10/TGFB/S100A9/MMP2/MMP9*. In addition, the formation of distant intestinal metastasis observed at the sites remote from the primary tumor appears to be mediated by immunosuppressive microenvironment generated by M2 macrophage accumulation. While this immunosuppressive microenvironment might as a key metastatic driver, alternative mechanisms underlying skip metastasis remain to be systematically investigated.

Primary tumors can promote metastasis by recruiting bone marrow-derived cells to distant organs and establishing supportive metastatic environments via tumor-derived soluble factors such as exosome 3, 34. However, the effect of exosomes and the exact mechanisms of the local invasion of primary tumor cells into adjacent tissues remain unclear. Hypoxia stimulates exosome generation and HIF2A expression 35, 36. Our study revealed that tumor-derived exosomes under hypoxic condition carrying *HIF2A* facilitated pre-invasive intestinal niche formation by inducing fibroblasts to secrete CXCL12, which supports M2 macrophage enrichment in peritumor tissues. *HIF2A* depletion or overexpression in primary CRC cell-derived exosomes influences pre-invasive niche initiation *in vivo*. The expression of the signaling molecules HIF2A/CXCL12/CXCR4 is associated with M2 macrophage marker CD163 expression in peritumor tissues of patients with CRC, and they are also associated with poor prognosis. Consistent with these results, hypoxia induces the formation of an immunosuppressive microenvironment 35, 36.

In our study, we aimed to clarify that HIF2A communication in exosomes primarily occurs through the mRNA form, utilizing RNase treatment in coculture experiments and fluorescence *in situ* hybridization (FISH) to confirm our hypothesis. This concept of exosome-mediated mRNA delivery is supported by recent reports of extracellular vesicles carrying *MMP1* mRNA 37, and tumor exosomal RNAs promoting dissemination 38. Some studies have shown that under normoxic conditions, HIF-2α is hydroxylated and degraded rapidly; under hypoxic conditions, HIF-2α becomes stabilized, rapidly accumulates in cells, dimerizes with HIF-1β, and thus translocates into the cellular nucleus, activating hypoxia response genes by binding to the hypoxic response element of its target genes 39. Chronic inflammation, a primary cause of colon cancer formation, characterizes the intestinal microenvironment in CRC patients, which is chronically inflamed and has been reported as hypoxic 40, 41. Hypoxia-inducible factors were detected in peritumor tissues 42. Our results also showed that HIF-2α expression in peritumor tissues was lower than in paired tumor tissues but higher than in normal tissues. Fibroblasts cultured with exosomes were maintained under hypoxic conditions, and HIF2A was stably expressed at both the mRNA and protein levels. Therefore, the published articles, in conjunction with our results, indicate that tumor cell-derived exosomes transmit *HIF2A* mRNA to peritumoral fibroblasts, which is then translated into HIF-2α protein, forms a heterodimer with HIF-1β, enters the cellular nucleus, and induces CXCL12 expression.

## Conclusions

In conclusion, exosome-mediated *HIF2A* derived from primary tumor activates peritumoral fibroblasts to secrete CXCL12, thus preparing 'soil' enriched with CXCR4^+^ M2 macrophages for future invasion of cancer cell. This signaling pathway, comprising exosome *HIF2A* and the CXCL12/CXCR4 axis, illustrates a novel mechanism involved in CRC progression.

## Methods and materials

### Study design

To explore the mechanisms responsible for local intestinal intestine invasion of CRC, we analyzed the fractions of 22 immune cell types from The Cancer Genome Atlas (TCGA) and found that the expression of M2 macrophages in the peritumor tissues was higher than that in the tumor tissues. Then, a total of 145 patients with CRC and 10 patients with intestinal perforations or benign lesions were randomly enrolled. M2 macrophages increased in the peritumoral intestinal tissues of patients with CRC and were associated with intestinal invasion of the primary tumor. Furthermore, PCR screening and bioinformatics analysis were used to explore the regulatory mechanisms and found that CXCL12 is highly expressed in CRC peritumor tissues, is responsible for recruiting monocytes to peritumoral sites and polarizing them into M2 macrophages by binding to CXCR4. HIF2A-exosome from primary CRC stimulated CXCL12 expression in peritumoral fibroblasts and induced M2 accumulation and tumor cell invasion, which was verified by an orthotopic CRC model of wild-type mice and macrophage-specific *Cxcr4* knockout mice. Finally, clinical data suggest that massive M2 macrophage infiltration and high expression of the signaling molecules CXCL12/CXCR4/HIF2A, predicted intestinal invasion and poor prognosis in patients with CRC.

### Clinical sample selection

Tissue and peripheral blood samples were obtained from patients with CRC, benign lesions, or intestinal perforation in the Department of Anorectal Surgery of the First Affiliated Hospital of Zhengzhou University (Zhengzhou, China). Normal intestinal tissues (N) (n = 10) were collected from patients with benign lesions or intestinal perforations. Tumor tissues (T) (n = 60) and paired peritumoral intestinal tissues (PT) (n = 60) were collected from patients with CRC. Paraffin sections of tissue samples from patients with CRC (n = 85) were obtained from the Pathology Department, and the clinicopathological data for CRC patients are listed in Supplementary table 1. All patients were pathologically diagnosed using tissue samples and did not receive any therapeutic intervention such as chemotherapy or radiotherapy. All patients were histologically diagnosed with CRC. These patients were staged according to the UICC-TNM classification. The samples included in this study were approved by the local ethics committee (ethics approval number: Science-2010-LW-1213), and informed consent was obtained from each patient with available follow-up information.

### Orthotopic animal tumor model studies

Specific pathogen-free (SPF), 6-week-old female BALB/c mice were purchased from Vital River Laboratory Animal Technology Co., Ltd. (Beijing, China) and randomly divided into N groups. We resuspended 5 ×10^5^ luciferase-CT-26 in 25 μL of Hank's Balanced Salt Solution (HBSS) (Invitrogen), mixed with 25 μL of Matrigel (BD Bioscience), and injected into the submucosal layer of the cecum using 29-gauge needles. Intestinal invasion was monitored weekly by *in vivo* bioluminescence imaging in the 1-, 2-, and 4-week treatment groups. Infiltration of immune and tumor cells in the peritumor intestine was detected by HE staining, immunofluorescence, and flow cytometry.

To evaluate the influence of macrophages or CXCR4 on tumor invasion, chlorophosphate liposomes or AMD3100 (a CXCR4 inhibitor, which selectively block the CXCR4 through interference with the interaction of CXCR4 with its natural ligand CXCL12) were administered intraperitoneally on days 8, 12, and 16 after the orthotopic injection of Luc-CT26 cells.

An adoptive-transfer mouse model was used in the present study. CD11b^+^CXCR4^+^ and CD11b^+^CXCR4^-^ cells (2 × 10^6^ cells) purified from mouse bone marrow were transferred intravenously 8 days after Luc-GFP-CT26 cells were orthotopically injected.

C57BL/6-Tg(csf1r-cre) 1Mnz/J mice were purchased from the Jackson Laboratory and maintained by Xinxiang Medical University (China). *Cxcr4^fl/fl^* mice (Stock No: NM-CKO-200156) were purchased from Shanghai Model Organisms (Shanghai, China). Mice were housed in a specific pathogen-free (SPF) facility in 12 h/12 h light/dark cycle at 18-24 ℃ and 30-70% of humidity range. These two types of mice were crossed to obtain *Csf1r^cre^Cxcr4^fl/fl^* mice. Orthotopic CRC murine models were generated by inoculating Luc-GFP-MC38 cells (2 × 10^6^) into the cecal wall. Tumor burden was evaluated using *in vivo* luminescence.

*HIF2A*-silenced exosomes, *HIF2A*-overexpression exosomes or vector exosomes were intraperitoneally injected every 2 days starting from day 2 after Luc-CT26 cells were orthotopically injected.

Tumor progression was monitored using *in vivo* bioluminescence imaging. The tumor weight and size were measured after the mice were sacrificed. Tissue samples were collected for immunofluorescence and flow cytometry analyses.

### Statistical analysis

Data analysis was performed using GraphPad Prism 10 software. Student's t-test was used for comparisons of the means between the two groups, two-way ANOVA was applied to determine the comparison among groups in a time course and the Kaplan-Meier test was used for survival rates. Pearson analysis was used for correlation analysis between the two indicators. Chi-square tests were used to analyze the correlation between molecular expression and clinical parameters. Statistical significance was set at p < 0.05.

## Supplementary Material

Supplementary methods, figures and tables.

## Abbreviations

CRC: colorectal cancer
BMDCs: bone marrow-derived cells
TAMs: tumor-associated macrophages
HIFs: hypoxia-inducible factors
TCGA: The Cancer Genome Atlas
PT: peritumoral intestinal tissues
SUP: supernatants
IRSs: immunoreactive scores
