# Supplementary material for: Tumor Exosomal HIF2A Induce Peritumoral M2 Macrophages Accumulation to Facilitate Intestinal Invasion in Colorectal Cancer
Source: Theranostics. 2025 Jul 2;15(15):7709–25. doi: 10.7150/thno.113190 (PMC12316027; doi:10.7150/thno.113190)
Supplement: Supplementary file 1 — Supplementary methods, figures and tables. [file thnov15p7709s1.pdf]

Supplementary methods and materials

Immunohistochemistry and immunofluorescence

Immunohistochemistry was performed using tissue samples fixed in formalin and embedded in paraffin-embedded slices. Immunofluorescence analysis was performed using frozen tissue samples and freshly fixed cells. The antibodies used in this research were as follows: anti-CD14 (1:300; Abcam), anti-CXCL12 (1:300; Abcam), anti-CD206 (1:300; Abcam), anti-CD163 (1:300; Abcam), anti-CD326 (1:300; Abcam), anti-CXCR4 (1:300; Abcam), anti-vimentin (1:300; Abcam), anti-HIF-2α (1:300; Abcam), anti-CD45 (1:300; servicebio), anti-F4/80 (1:300; servicebio) and anti-GFP (1:300; Abcam). The immune response score (IRS) was calculated as follow. Three fields of view per sample were imaged using immunohistochemistry, and the intensity of immunostaining was considered. Estimation of the percentage scoring of immunoreactive cells was as follows: 0 (< 10%), 1 (10–40%), 2 (40–70%), and 3 (> 70%). Staining intensity was visually scored by three different individuals and stratified as follows: 0 (negative), 1 (yellowish), 2 (light brown), and 3 (dark brown). IRS were obtained by multiplying the two items by the total score and ranged from 0 to 9. Protein expression levels were further analyzed by classifying IRS values as low (based on an IRS value ≤ 5) and as high (based on an IRS value > 5). Frozen samples were visualized under a fluorescence microscope (Olympus, IX71, Japan). For the analysis of immunofluorescence images, the rate of positive cells was calculated using Aipathwell software (Servicebio) [43].

Isolation of Intestinal Mucosa Cells

Intestinal mucosal tissue was obtained and initially rinsed with PBS to clear the intestinal contents. Subsequently, the tissue was immersed in 10 mL of D-HankS incubation solution enriched with 1 mmol/L dithiothreitol (DTT), 1 mmol/L ethylenediaminetetraacetic acid (EDTA), and 10% fetal bovine serum without magnesium and calcium ions for 10 min to eliminate mucus and epithelial cells. The tissue was then crushed using ophthalmic scissors and transferred into a 1640 medium

solution at a ratio of 5 mL per square centimeter of tissue. This medium was supplemented with 1 mg/mL of collagenase and 10% calf serum. The mixture was incubated on a 37 °C shaking platform set at 100 rpm/min for 20 min to facilitate cell digestion. The cell suspension was strained through a 300 mesh sieve to separate the cells.

Flow cytometry analysis

Mononuclear cells from tissues and PB were isolated and stained for 30 min at 4 °C using saturating concentrations of the following antibodies: anti-human CD14, CD163, CXCR4 and 7-AAD antibodies and anti-mouse CD11b, F4/80, CXCR4 and CD206 (BioLegend, USA). Flow cytometric analysis was performed using a BD FACSCanto II (USA) instrument. The mononuclear cells in tissue were obtained and the dead cells were removed by labeling 7-AAD. The proportion of M2 macrophages in patients was evaluated by the percentage of $CD163^+$ cells in the $CD14^+$ cells. The proportion of M2 macrophages in mice means the percentage of $CD206^+$ cells in the $F4/80^+$ cells. The proportion of $CXCR4^+$ cells means the percentage of $CXCR4^+$ cells in M2 macrophages.

Transwell Assay

To block monocyte migration, $CD14^+$ cells were pre-treated with AMD3100 (10 nM, Selleck) for 2 h. Then, treated or untreated $CD14^+$ cells were seeded in the upper chambers with a 5 μm pore size. Supernatants of fibroblasts or recombinant human (rh) CXCL12 (PeproTech, USA) and anti-CXCL12 (100 ng/mL, Abcam) were added to the lower chambers. The migrated cells in the lower chambers were counted after 12 h.

Western blot

Total protein was extracted with RIPA lysis buffer supplemented with a protease inhibitor cocktail (Sigma-Aldrich, P8340) and phosphatase inhibitor cocktail 2 (Sigma-Aldrich, P5726). Antibodies used in this research were as follows: anti-phosphor-MAPK (1:1000; Cell Signaling technology, USA), anti-phosphor-ERK (1:1000; Cell Signaling technology, USA), anti-phosphor-PI3K (1:1000; Cell Signaling technology,

USA), anti-phosphor-AKT (1:1000; Cell Signaling technology, USA), anti-phosphor-STAT3 (1:1000; Cell Signaling technology, USA), anti-phosphor-P65 (1:1000; Cell Signaling technology, USA), anti-HIF-2α (1:1000; Cell Signaling technology), and anti-β-actin (1:3000; Cell Signaling technology) as control. These primary antibodies were detected using a horseradish peroxidase-conjugated anti-IgG antibody (1:1000; Cell Signaling Technology).

Enzyme linked immunosorbent assay

The concentration of CXCL12 in the fibroblast supernatant was measured using enzyme linked immunosorbent assay (ELISA) (R&D Systems Inc., Minneapolis, MN, USA), according to the manufacturer's instructions.

Quantitative real-time PCR

Total RNA was extracted with TRIZOL Reagent, and cDNA was prepared by reverse transcription according to previous studies (reference). (Invitrogen, Carlsbad, CA, USA). Quantitative real-time PCR was performed using SYBR (BioConnect Services Pty Ltd., Melbourne, Australia) and GAPDH was used as an internal control. The primers used are listed in Supplementary table 2. The relative expression of genes was calculated using 2-ΔΔCt method.

Multiplex assay

The levels of cytokines in the conditioned medium of $CD163^{+}CD14^{+}$ cells from tumor and peritumor tissues of patients with CRC were analyzed using a multi-analyte flow assay kit (BioLegend, USA), including 13 human cytokines, according to the manufacturer's instructions.

Isolation of monocytes

Human CD14 magnetic beads (Miltenyi Biotec) were used to isolate $CD14^{+}$ monocytes from PBMC, according to the manufacturer's instructions. $CXCR4^{+}CD11b^{+}$ and $CXCR4^{-}CD11b^{+}$ cells were sorted from mouse bone marrow using a MoFlo XDP flow

cytometer (Beckman Coulter). The positivity rate of cells after purification was > 90%.

Polarization of M2 macrophages

$CD14^+$ monocytes sorted from peripheral blood of healthy people were stimulated with rhM-CSF (20 ng/mL), rhCXCL12 (10 ng/mL) (PeproTech, USA), or supernatant of fibroblast for 7 days in 37 °C, 5% CO_2. The supernatant was changed every 2 days. After 7 days, the cells were evaluated by morphological, phenotypic, and functional analyses.

Luciferase reporter assay

Putative binding sites for HIF-2α on the 3'-UTR of *CXCL12* were predicted by JASPAR. cDNAs containing partial CDS sequence near stop codon and full-length 3'UTR of *CXCL12* were cloned into pGL4.10-Basic vector (Promega) which was comprised of firefly luciferase. Pre-treated 293T cells were seeded into 24-well plate followed by transfecting with vectors, either expressing wild-type or mutant *CXCL12* promoter using Lipofectamine 3000 (Thermo Fisher Scientific). After 24–36 h, cells were harvested to access the luciferase activity using Dual Luciferase Reporter System (Promega). The experiments were repeated three times. The data are presented as the fold-change relative to the control group.

ChIP-qPCR assays

1×10^5 293T cells cultured under hypoxia condition were performed using Simple ChIP Enzymatic Chromatin IP Kit #9003 (Cell Signaling Technology) according to the manufacturer's protocol. Immunoprecipitation was performed using an HIF-2α antibody (Thermo Fisher, MA5-44201) or with rabbit IgG as a negative control. The immunoprecipitated DNA fragments were purified and extracted to RT- PCR using CXCL12 promoter–specific primers. Primers used for amplifying binding fragment of CXCL12 promoter are: forward: GGCTCCGTGGGAAGAGTTTT; reverse: GATCTGCGGGAATGAGACCC.

Isolation and purification of exosomes

HCT116/SW480/CT-26 cells were plated in T125 flasks supplemented with 1640 medium containing 10% exosome-free FBS (removed by ultracentrifugation) for 48 h, and supernatant was collected. The supernatant was centrifuged at 300 *g* for 10 min, followed by 2000 *g* for 20 min to remove cells, 20000 *g* for 10 min to remove dead cell, 1000 *g* for 30 min to remove cell debris. Next, the supernatant was filtered using a 0.2 μm filter and then ultracentrifuged at 120000 *g* for 2 h at 4 °C (CP80WX, HIITACHI, Japan). The supernatant was discarded and pellets were washed with PBS and ultracentrifuged at 120000 *g* for 1 h at 4 °C. Exosomes were collected from the sediments for further experiments.

Identification of exosomes

Transmission electron microscopy was used to verify the presence of exosomes. Exosomes were resuspended in PBS and pipetted 100 μL on a 200 mesh copper grid. Thereafter, using 3% phosphotungstic acid to stain the exosomes for 10 min at 25 °C and dry for 12 h for TEM examination (JEOL USA, Inc., Peabody, MA, USA). For nanoparticle tracking analysis, 1 mL of PBS containing exosomes was analyzed using a ZETASIZER Nano series-Nano-Z (Shanghai Yuanzi Biotechnology Co., Ltd, Shanghai, China). For the analysis of exosome protein markers, exosomes were prepared in ice-cold RIPA buffer (Solarbio) and analyzed by western blotting as described previously. Anti-CD9, -TSG101 (Abcam), and anti-GAPDH antibodies were used at a 1:1000 dilution.

Bioinformatics analysis

RNA sequence data and clinical parameters of CRC were downloaded from the UCSC Cancer Browser (https://genome-cancer.ucsc.edu). The GSE21510 datasets were downloaded from GEO (https://www.ncbi.nlm.nih.gov/geo/). CIBERSORT" software was used to analyze immune cell infiltration from TCGA database. Upstream signaling of CXCL12 was screened using the PROMO website (https://alggen.lsi.upc.es/cgi-

bin/promo_v3/promo/promoinit.cgi?dirDB=TF_8.3).

Exosome uptake analysis

Exosomes were stained using a PKH26 kit (Sigma-Aldrich, St. Louis, MO, USA) according to the manufacturer's instructions. HCT116 cells were resuspended at 1 × 10^7/mL with diluent C and incubated with 4 μL of PKH26 ethanol solution for 1–5 min. Thereafter, 1 mL of fetal bovine serum was added to stop the reaction. After centrifugation, HCT116 cells were cultured with exosome-free serum for 2 days and PKH26-stained exosomes were collected. Fibroblasts were incubated with PKH26-stained exosomes for 12 h and then fixed for observation of PKH26 fluorescence using a confocal microscope (IX71; Olympus, Tokyo, Japan).

RNA-fluorescence in situ hybridization (Fish)

HIF2A mRNA levels were measured using RNA-fluorescence in situ hybridization (RNA-FISH). Briefly, HSF cells were plated on round cover slips prior to use. The cells were co-cultured with exosomes in a tri-gas incubator with an oxygen concentration of 1% for 12 h. The cells were then fixed with a FISH fixative solution (ServiceBio) for 15 min, followed by the addition of protease K (20 μg/mL) for digestion at 40 °C for 5 min. A pre-hybridization solution was applied and incubated at 37 °C for 1 h. The hybridization solution containing the probe was incubated overnight at 40 ℃, then washed with 2 × SSC for 10 minutes, followed by 1 × SSC twice for 5 min each at 37 °C, and finally with 0.5 × SSC at room temperature for 10 min. A pre-branched probe was added, and the slides were incubated in a humid chamber for hybridization at 40 °C for 45 min. The slides were sequentially washed with 2 × SSC, 1 × SSC, 0.5 × SSC, and 0.1 × SSC pre-warmed to 40 °C for 5 min each. The hybridization solution containing the signal probe was diluted at a ratio of 1:200 and incubated at 42 °C for 3 h. The slides were then sequentially washed with 2 × SSC for 10 min, 1 ×

SSC twice for 5 min each at 37 °C, and 0.5 × SSC at 37 °C for 10 min. DAPI staining solution was added to the slides in the dark for 8 min, followed by rinsing. An anti-fluorescence quenching sealant was applied to seal the slides.

RNA-sequencing

The 50 mL supernatant of 5 × 10^6 HCT116 cell per plate were collected, and centrifuged at 3000 rpm for 10min to remove impurities such as cellular debris. Three replicates were performed for per group. Exosomes were enriched and total RNA was extracted from isolated exosomes using TRIzol™ reagent followed with silica-membrane column purification (miRNeasy Mini Kit) to maximize yield and quality. RNA-sequencing was performed on an Illumina HiSeq platform.

Supplementary figure and legends

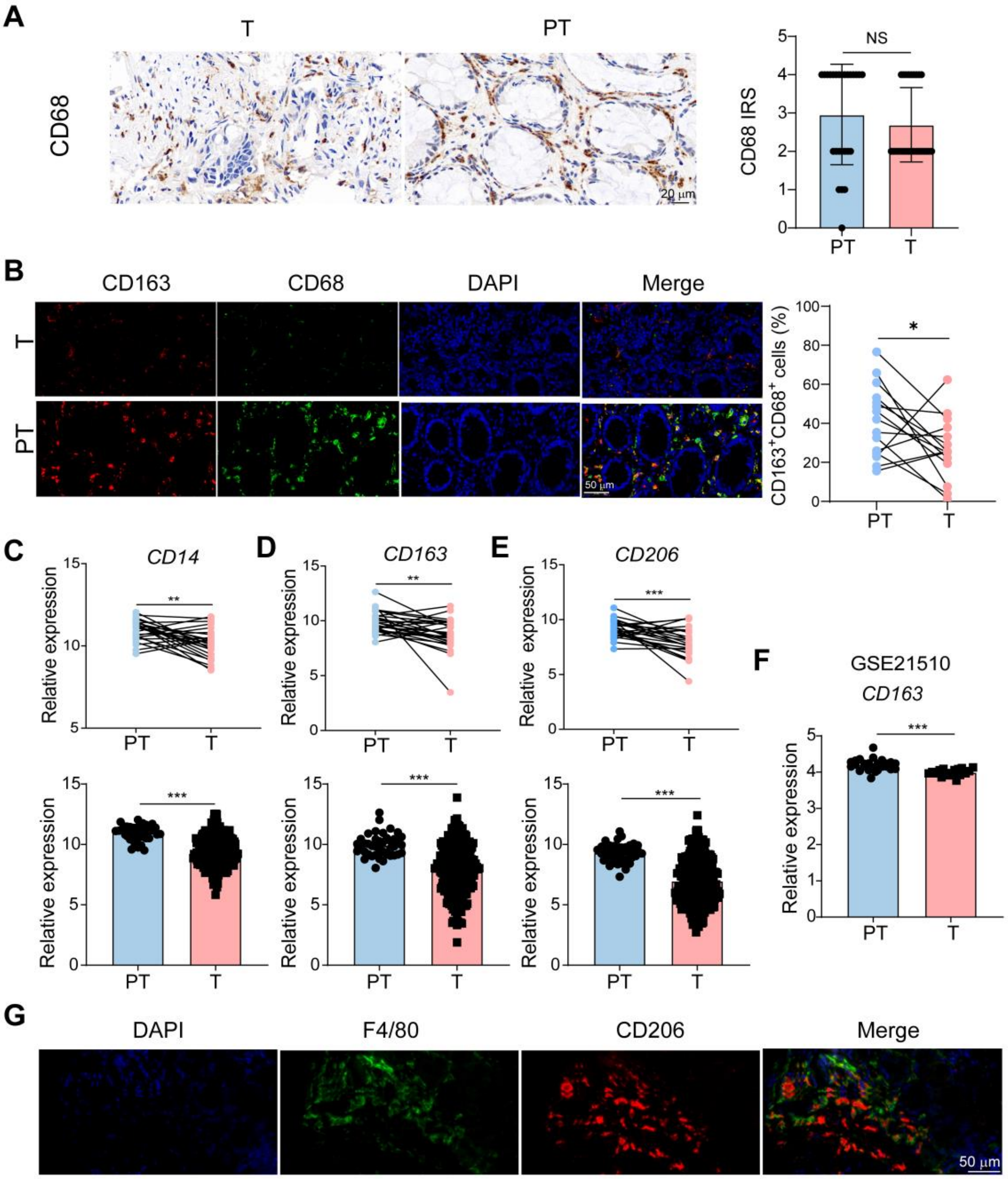

Supplementary Fig.1 High expression levels of M2 macrophage in peritumor tissues of CRC. A. Immunohistochemical profiles of CD68 in PT and T from patients with CRC under microscopy (Left); IRS of CD68 in PT and T (Right). **B.** Representative images of IF for CD68^{+} (green) and CD163^{+} (red) macrophages in peritumor and tumor tissues. The proportion of CD163^{+}CD68^{+} M2 macrophages in CD68^{+} cells were evaluated by IF analysis. **C-E.** Paired-samples *t* test (upper, n = 26) and unpaired *t* test (Below, n = 50) were used to analyze *CD14/CD163/CD206* expressions in T and paired PT of 26 CRC patients from TCGA dataset. **F.** *CD163* expression in CRC patients from GSE21510 dataset was detected by unpaired *t* test (n = 25). **G.** Representative images of immunofluorescence for F4/80^{+} (green) and CD206^{+} (red) macrophages in peritumor tissue of mice. P values were determined by

two-tail Student's t test; Data were shown by mean ± s.e.m. * $P < 0.05$, ** $P < 0.01$, ***$P < 0.001$; NS, no significance.

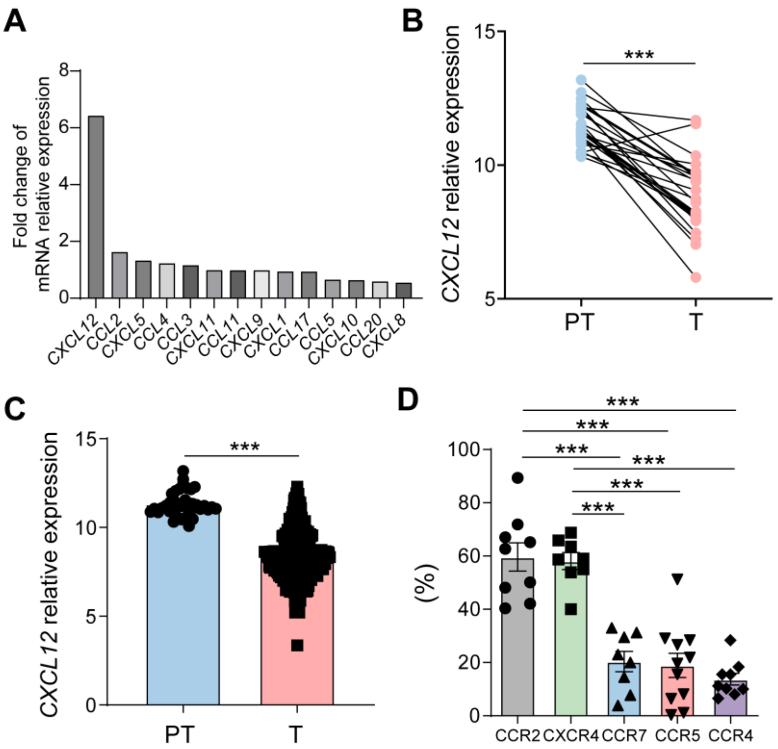

Supplementary Fig.2 High CXCR4 expression levels in monocytes from peripheral blood of patients with CRC.

A. The fold change of chemokines' mRNA relative expression in PT vs T. **B.** Paired-samples t test was used to analyze *CXCL12* expressions in T and paired PT of 26 patients with CRC from TCGA dataset. **C.** *CXCL12* expressions in 50 patients with CRC from TCGA dataset were detected by unpaired *t* test (n = 50). **D.** The proportions of CCR2, CCR4, CCR5, CCR7, and CXCR4 positive $CD14^{+}$ cells in peripheral blood were examined by flow cytometry. NC, isotype negative control; P values were determined by Two-tailed Student's unpaired t test. Data were shown by mean ± s.e.m. *** $P < 0.001$.

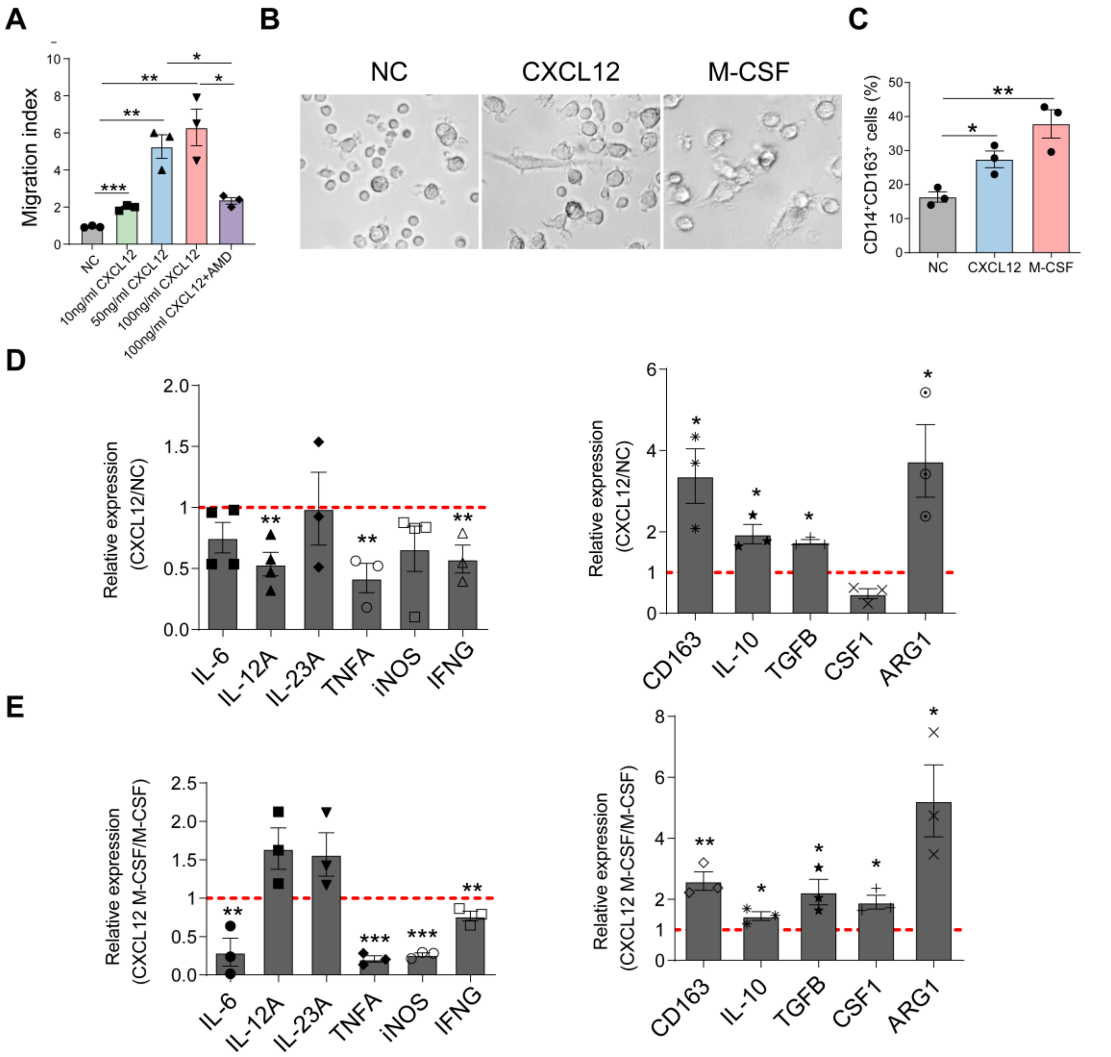

Supplementary Fig.3 CXCL12 mediates monocytes migration and polarization. A. Migration of CD14^{+} cells co-cultured with recombinant human CXCL12 (10, 50, and 100 ng/mL) and/or AMD3100 was analyzed by Transwell assay. **B.** Morphological features of monocytes under M-CSF or CXCL12 stimulation were detected by microscopy. **C.** The proportions of CD163^{+} M2-like macrophages in CD14^{+} cells were detected by flow cytometry. **D, E.** The expressions of M1- and M2-related genes were detected in CD14^{+} cells co-cultured with recombinant protein CXCL12 (10 ng/mL) and/or M-CSF (10 ng/mL) by qPCR. P values were determined by Two-tailed Student's unpaired t test in panel A, C, D, E; Data were shown by mean ± s.e.m. * $P < 0.05$, ** $P < 0.01$, *** $P < 0.001$.

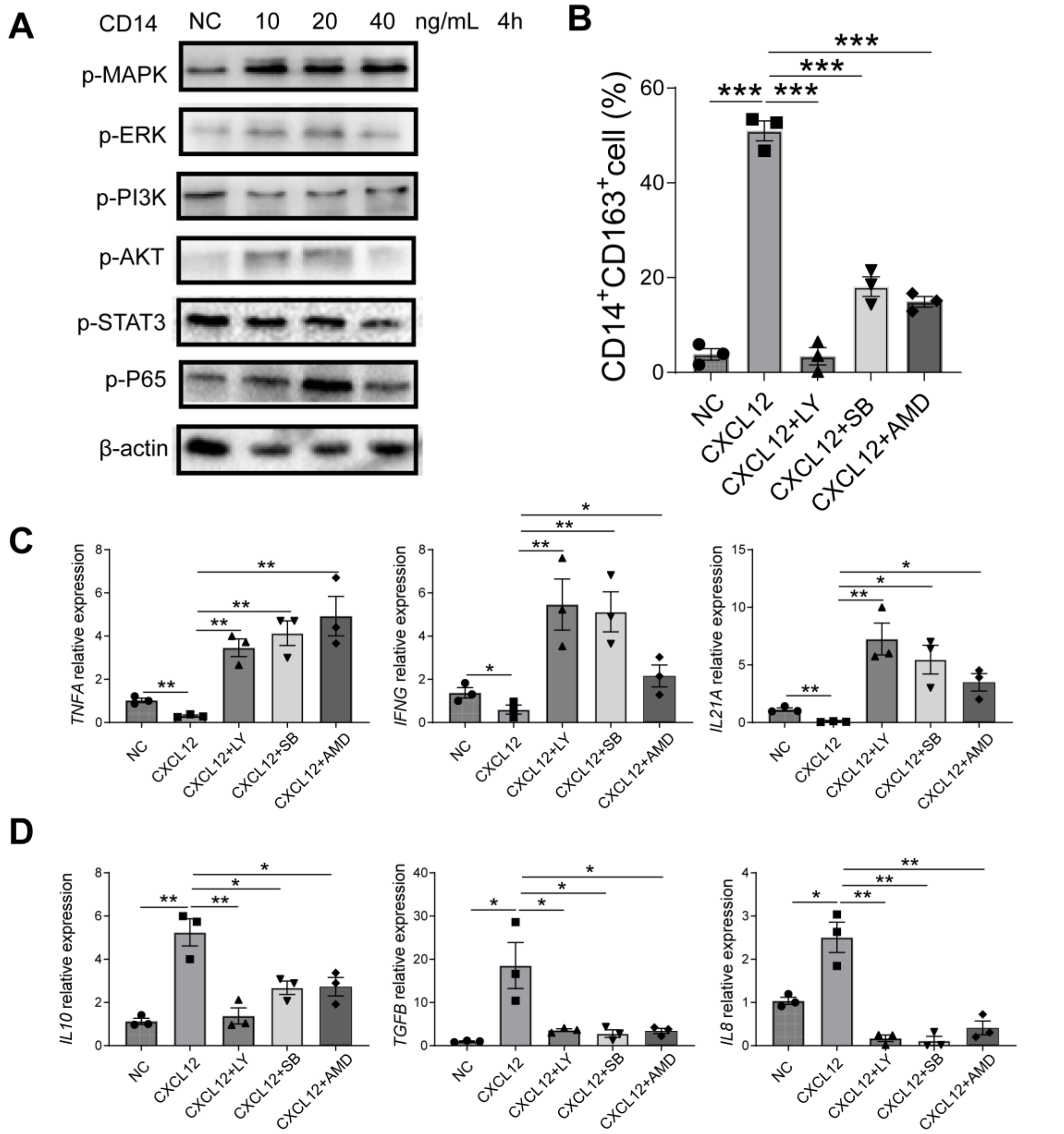

Supplementary Fig.4 CXCL12 stimulates monocyte polarization via CXCL12/ CXCR4/MAPK/ERK pathway. A. The signaling activation in $CD14^+$ cells after treatment with recombinant protein CXCL12 (10 ng/mL) was assessed by western blotting. **B.** The proportions of $CD163^+$ M2 macrophages in $CD14^+$ cells were detected by flow cytometry. **C and D.** The expressions of M1- and M2-related genes were detected by qPCR. P values were determined by Two-tailed Student's unpaired t test in panels B, C, D; Data were shown by mean ± s.e.m.* $P < 0.05$, ** $P < 0.01$, *** $P < 0.001$.

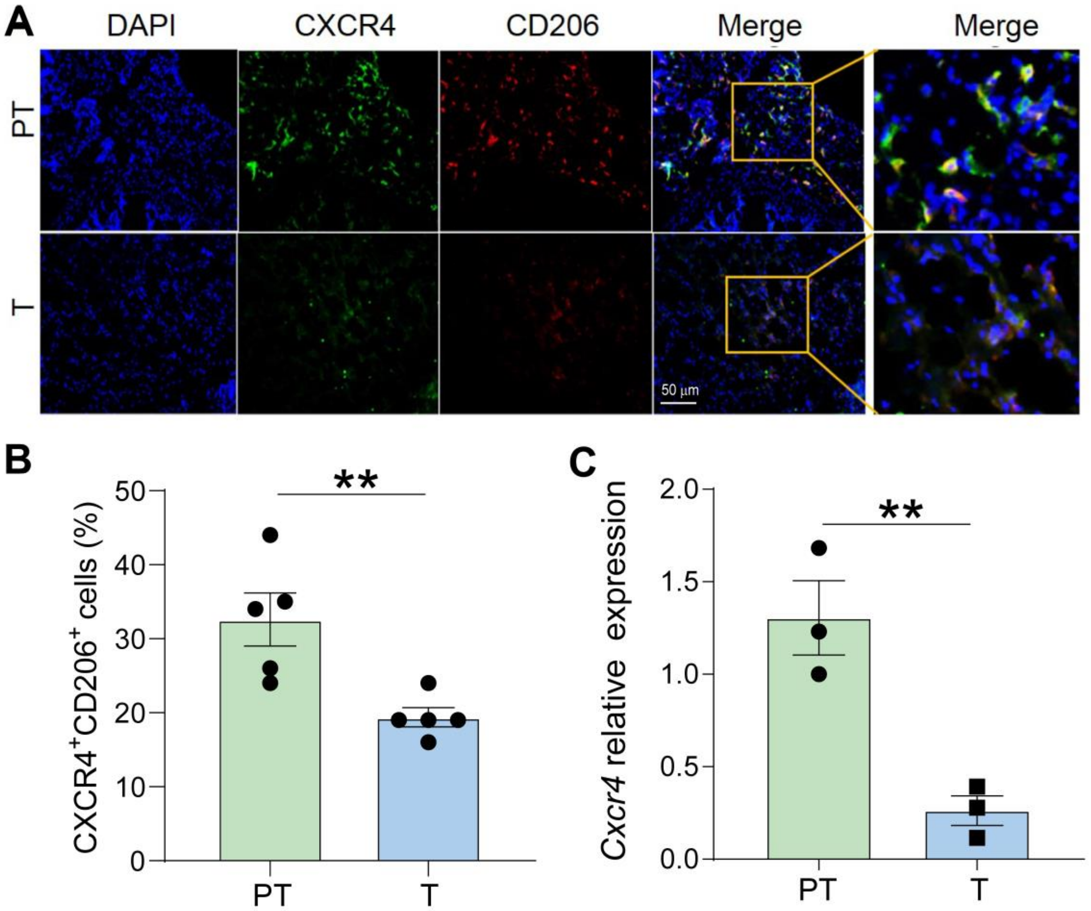

Supplementary Fig.5 CXCR4 was highly expressed in peritumoral M2 macrophages. A, B. Representative images of IF for CXCR4$^+$ (green) and CD206$^+$ (red) M2 macrophages in PT and T from orthotopic mice. CXCR4$^+$CD206$^+$ cell percentage were evaluated by IF analysis. **C.** The mRNA level of *Cxcr4* was quantified in purified CD206$^+$CD11b$^+$ M2 macrophages. P values were determined by Two-tailed Student's t test in panels B, C; Data were shown by mean ± s.e.m. * $P < 0.05$, ** $P < 0.01$.

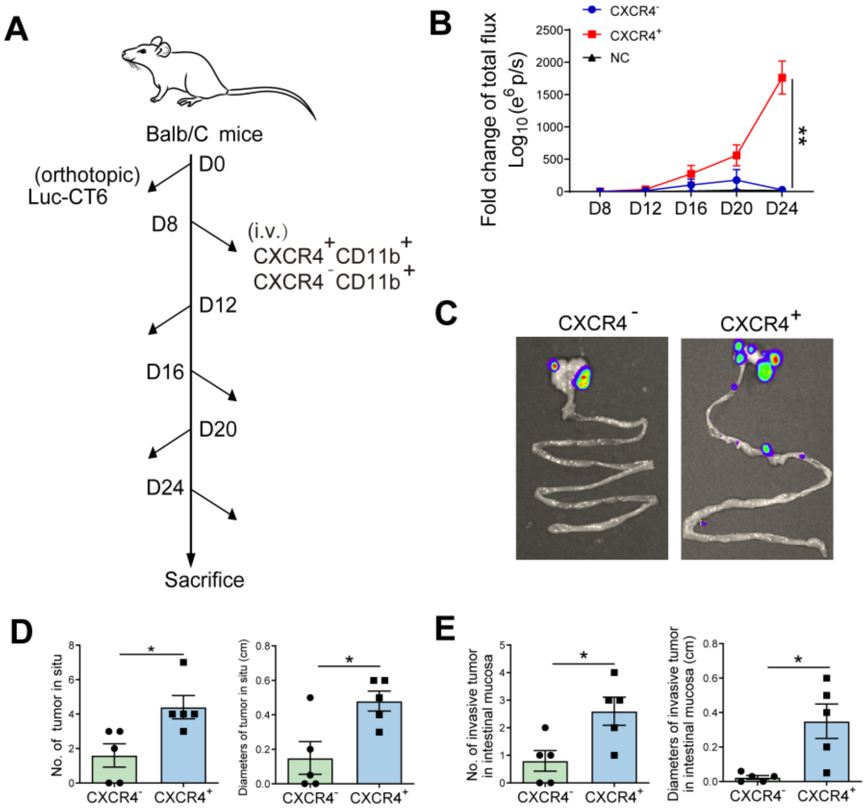

Supplementary Fig.6 CXCL12 mediates primary tumor growth and invasion depending on CXCR4. A. Schematic illustration for the $CD11b^{+}CXCR4^{+}$ and $CD11b^{+}CXCR4^{-}$ cells (2×10^{6} cells) purified from mouse bone marrow cell transfer intravenously at 8 days after Luc-GFP-CT26 cells were injected orthotopically. Tumor evolution was monitored every 4 days using *in vivo* bioluminescence imaging. Primary tumor and intestinal invasion were detected at day 24. **B.** Quantitative photon counting analysis of tumor progression by an *in vivo* imaging system. **C.** Images of primary tumor in the cecum and invasive tumor in the adjacent colon by an *in vivo* imaging system. **D and E.** The diameters and numbers of primary tumor and invasive tumor in intestinal tissues were evaluated. P values were determined by two-way ANOVA in panel B, or two-tailed Student's t test in panel D, E; Data were shown by mean ± s.e.m. * $P < 0.05$.

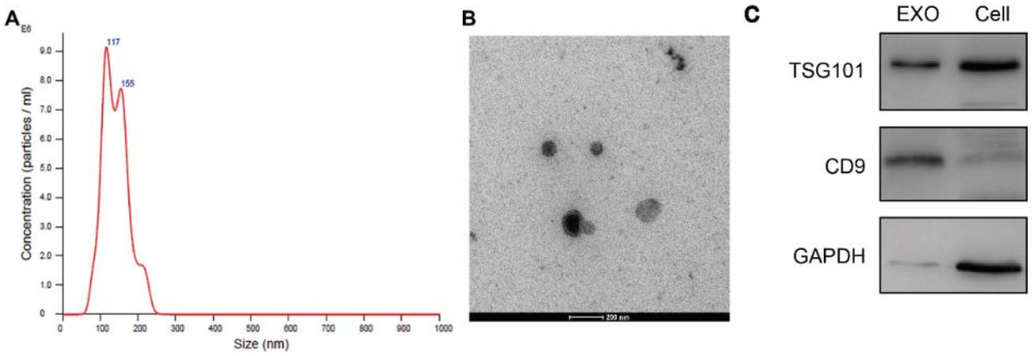

Supplementary Fig.7 Characterization of isolated exosomes. A. Size distribution was determined using NanoSight. **B.** Electron micrographs of exosomes isolated from HCT116 cells. Exosomes of 30–150 nm in diameter were shown. The scale bar

indicates 200 nm. **C.** Western blot was performed for the indicated proteins.

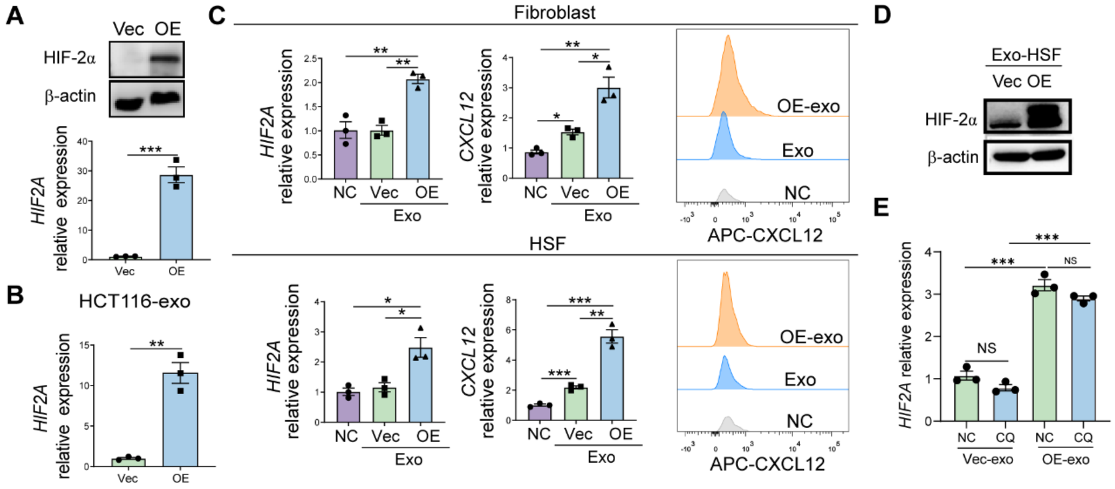

Supplementary Fig.8 Overexpression *HIF2A* in tumor cell induces the increase of HIF2A and CXCL12 expression in fibroblasts through *HIF2A* mRNA-exosome. A. *HIF2A* was examined by qPCR and HIF-2α by western blotting in HIF2A over-expression HCT116 cells (OE: OE-*HIF2A*) and control (Vec: Vector). **B.** *HIF2A* was examined by qPCR in OE-*HIF2A*-HCT116- or Vector-HCT116-derived exosome. **C.** *HIF2A* mRNA and CXCL12 expressions were evaluated in HSF and primary fibroblasts treated with or without OE-*HIF2A*-HCT116- or Vector-HCT116-derived exosomes. **D.** HIF-2α expression on protein level in HSF treated with OE-*HIF2A*-HCT116- or Vector-HCT116-derived exosomes. **E.** *HIF2A* mRNA expression in HSF cocultured with OE-*HIF2A*-HCT116- or Vec-HCT116-derived exosomes in the presence or absence of CQ for 24 h. P values were determined by two-tailed Student's t test in panel A-C, E; Data were shown by mean ± s.e.m. ** P < 0.05, ** P< 0.01, *** P < 0.001; NS, no significance.

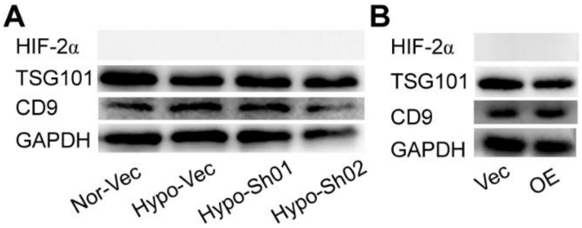

Supplementary Fig.9 HIF-2α protein were not expressed in exosomes from hypoxic tumor cells or sh-*HIF2A* cells or OE-*HIF2A* cells. HIF-2α expression on protein level in exosomes from sh*HIF2A*-HCT116- (**A**) and OE-*HIF2A*-HCT116- cells (**B**).

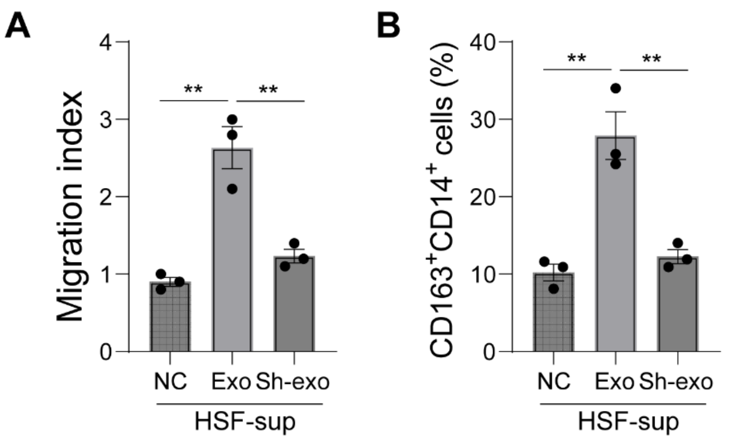

Supplementary Fig.10 The accumulation of M2 macrophages primarily depends on HIF2A in exosome. A. Migration of CD14^{+} cells co-cultured with HSF-supernatant, which were processed with Vector-HCT116- or Sh*HIF2A*-HCT116-derived exosomes under hypoxia (Exo or sh-exo) or Vector-HCT116-derived exosomes under normoxia (NC). **B.** The proportions of CD163^{+} M2 macrophages were detected in CD14^{+} cells incubated with HSF-supernatant, which be processed with exosome. Sup, supernatant. P values were determined by two-tailed Student's t test; Data were shown by mean ± s.e.m. ** P < 0.01.

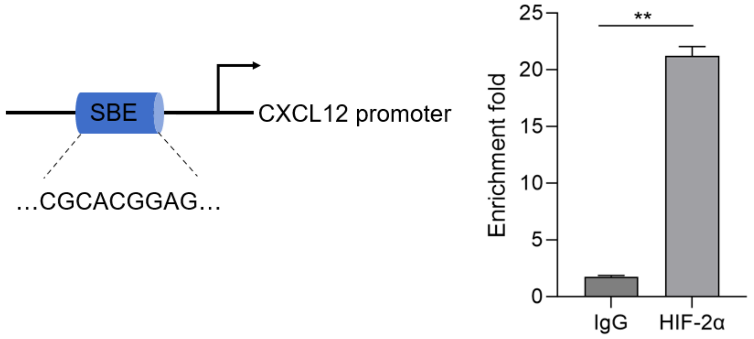

Supplementary Fig.11 ChIP-qPCR analysis reveals specific binding of HIF-2α to the promoter region of CXCL12. HIF-2α binding site on the CXCL12 promoter was predicted in the JASPAR database (Left). The binding of HIF-2α to the CXCL12 promoter was analyzed by ChIP assay (Right). N = 3, Student's t test. ** P < 0.05. Data were shown by mean ± s.e.m. ** P < 0.01.

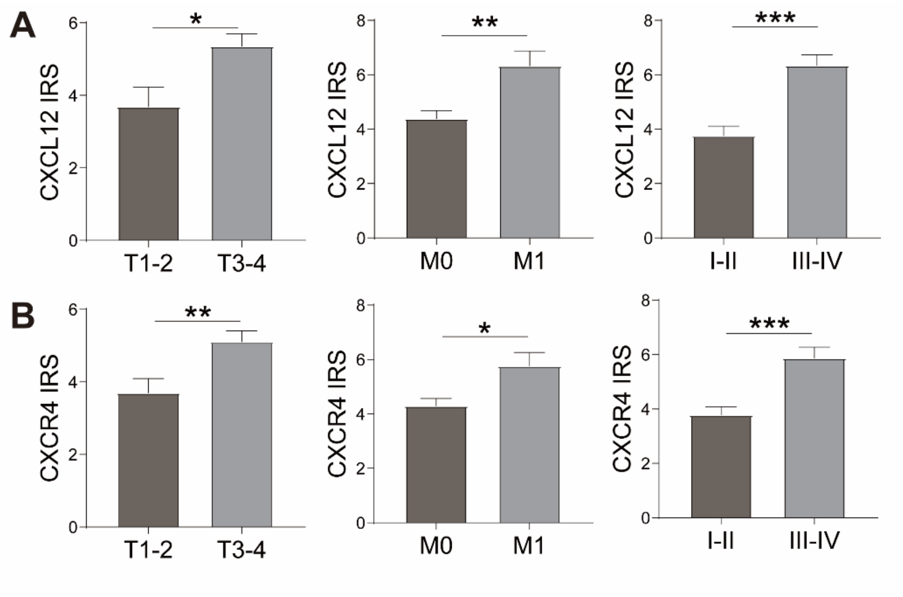

Supplementary Fig.12 The relation between CXCL12/CXCR4 expression and clinicopathological characteristics of patients with CRC. A and B. IRS of CXCL12/CXCR4 in PT from patients with CRC sub-grouped by TNM, T, and M stages. T1-2, tumor does not reach the subserous membrane; T3-4, tumor reaches or extends beyond the subserous membrane; M0, non-distant metastasis; M1, distant metastasis; I-II, early stage; III-IV, advanced stage). P values were determined by two-tailed Student's t test in panel A, B; Data were shown by mean ± s.e.m. * P < 0.05, ** P < 0.01, ***P < 0.001.

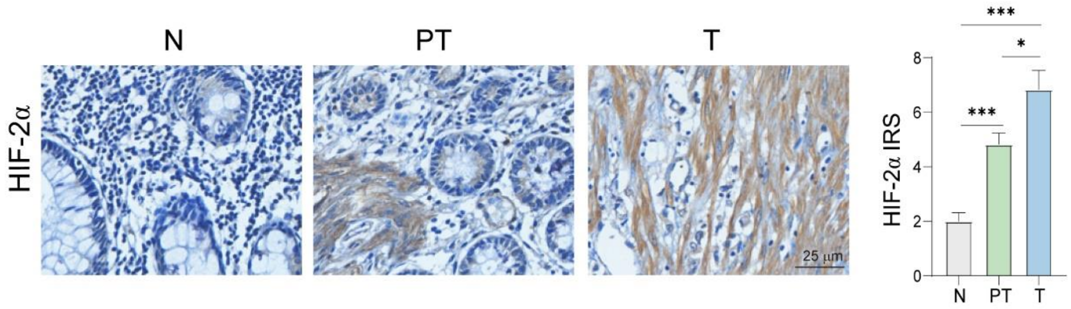

Supplementary Fig.13 The HIF-2α expression in the peritumor tissues was lower than that in the paired tumor tissues but higher than that in the normal tissues. Immunohistochemical profiles of HIF-2α in N, PT and T under microscopy (Left); IRS of HIF-2α in N, PT and T (Right). P values were determined by two-tailed Student's t test; Data were shown by mean ± s.e.m. * P < 0.05, ** P < 0.01, *** P < 0.001.

Supplementary Table 1.

Clinicopathological data for CRC patients

**Characteristics**	**CRC patients**	
	Number	%
**Gender**		
Male	41	48.2
Female	44	51.8
**Age (years)**		
<60	49	57.6
≥60	36	42.4
**Location**		
Colon	50	58.8
Rectum	35	41.2
**Pathological type**		
Adenocarcinoma	75	88.2
Others	10	11.8
**Differentiation**		
Well	71	83.5
Poor	14	16.5
**T stage**		
T1-2	33	38.8
T3-4	52	61.2
**N stage**		
N0	61	71.8
N1-3	24	28.2
**M stage**		
M0	70	82.4
M1	15	17.6
**TNM Stage**		
I-II	51	60.0
III-IV	34	40.0

Supplementary Table 2.

Quantitative RT-PCR primer sequences

Gene name	Forward primer	Reverse primer
GAPDH	GCACCGTCAAGGCTGAGAAC	TGGTGAAGACGCCAGTGGA
CCL2	CAGCCAGATGCAATCAATGCC	TGGAATCCTGAACCCACTTCT
CCL3	TCAGACTTCAGAAGGACACGG	CTGCATGATTCTGAGCAGGTG
CCL4	CTGTGCTGATCCCAGTGAATC	TCAGTTCAGTTCCAGGTCATACA
CCL5	CCAGCAGTCGTCTTTGTCAC	CTCTGGGTTGGCACACACTT
CCL11	CCCCTTCAGCGACTAGAGAG	TCTTGGGGTCGGCACAGAT
CCL17	AGCCATTCCCCTTAGAAAGC	CTGCCCTGCACAGTTACAAA
CCL20	TGCTGTACCAAGAGTTTGCTC	CGCACACAGACAACTTTTTCTTT
CXCL1	AGCTTTGTTTAAACATGGCC<br>CGCGCTGCTCTC	AGCTTTGTTTAAACCCCTTC<br>TGGTCAGTTGGATTTG
CXCL5	AGCTGCGTTGCGTTTGTTTAC	TGGCGAACACTTGCAGATTAC
CXCL8	GTTGTAGGGTTGCCAGATGC	TTCTCCCGTGCAATATCTAGG
CXCL9	CCAGTAGTGAGAAAGGGTCGC	AGGGCTTGGGGCAAATTGTT
CXCL10	GTGGCATTCAAGGAGTACCTC	TGATGGCCTTCGATTCTGGATT
CXCL11	CCTTGGCTGTGATATTGTGTGC	CCTATGCAAAGACAGCGTCCT
CD163	ACTTGAAGACTCTGGATCTGCT	CTGGTGACAAAACAGGCACTG
CD206	GGGTTGCTATCACTCTCTATGC	TTTCTTGTCTGTTGCCGTAGTT
TNFA	CTGTAGCCCATGTTGTAGCAAAC	GCTGGTTATCTCTCAGCTCCAC
TGFB	GCCAGAGTGGTTATCTTTTGATG	AGTGTGTTATCCCTGCTGTCAC
IFNG	TCGGTAACTGACTTGAATGTCCA	TCGCTTCCCTGTTTTAGCTGC
IL6	CAGTTCCTGCAGAAAAAGGC	AACAACAATCTGAGGTGCCC
IL10	GCTCCAAGAGAAAGGCATCTAC	GGGGTTGAGGTATCAGAGGTAAT
INOS	TTCAGTATCACAACCTCAGCAAG	TGGACCTGCAAGTTAAAATCCC
ARG1	GTGGAAACTTGCATGGACAAC	AATCCTGGCACATCGGGAATC
HIF2A	CGGAGGTGTTCTATGAGCTGG	AGCTTGTGTGTTCGCAGGAA
